# Transcranial High-Frequency Terahertz Stimulation Alleviates Anxiety-like Behavior in Mice via a Noninvasive Approach

**DOI:** 10.34133/research.0766

**Published:** 2025-08-08

**Authors:** Pan Wang, Chaoyang Tan, Wenyu Peng, Zekun Yan, Wenrui Jiang, Han Zhao, Huaxing Si, Jingchen Jia, Chunkui Zhang, Jian Wang, Yuchen Tian, Kun Chen, Yuefan Yang, Zhenyu Wu, Kangning Xie, Yuanming Wu, Mingming Zhang, Tao Chen

**Affiliations:** ^1^ Department of Anatomy and K.K. Leung Brain Research Centre, School of Basic Medicine, Fourth Military Medical University, 710032 Xi’an, China.; ^2^ Department of Biochemistry and Molecular Biology, School of Basic Medicine, Fourth Military Medical University, 710032 Xi’an, China.; ^3^ School of Physics and Information Technology, Shaanxi Normal University, 710119 Xi’an, China.; ^4^ Beijing Institute of Basic Medical Sciences, Beijing 100850, China.; ^5^ Department of Neurosurgery, The Hospital of 81st Group Army PLA, 075000 Zhangjiakou, China.; ^6^ School of Biomedical Engineering, Fourth Military Medical University, Xi’an 710032, China.

## Abstract

Terahertz waves, positioned between infrared and microwave frequencies, have important potential in various fields, but their potential for in vivo biomedical applications has mostly remained untapped. In the present study, we focused on testing the potential of noninvasive high-frequency terahertz stimulation (HFTS) as a treatment for anxiety in mice. Mice were subjected to acute restraint stress to induce anxiety and then clustered into anxiety-susceptible and anxiety-resilient groups using the *K*-means algorithm. We developed an anxiety phenotype prediction classifier utilizing the naïve Bayes algorithm to accurately categorize mice. Noninvasive HFTS was targeted at the anterior cingulate cortex across the skulls of anxiety-susceptible mice, resulting in a marked anxiolytic effect. The underlying mechanism of HFTS’s anxiolytic effect was subsequently elucidated through in vivo and in vitro electrophysiological and morphological methods, revealing that HFTS decreases the excitability of pyramidal neurons in the anterior cingulate cortex by enhancing voltage-gated K^+^ channel and leak K^+^ channel conductance. The study not only expands the potential applications of HFTS, particularly its noninvasive use, in the regulation of anxiety-like disorders but also introduces innovative methodologies and insights that have the potential to lay the groundwork for future research in the field of physical biomedicine.

## Introduction

Terahertz waves, situated between infrared and microwave frequencies, bridge the gap between photonics and electronics [1]. While terahertz waves have important applications in physics, environmental science, aerospace, and other fields, their biological effects and biomedical application remain largely unexplored [2–4]. Unlike optogenetics, which necessitates the delivery of virus-mediated light-controlled tools to affect the central nervous system [5], terahertz waves are label-free and do not require the introduction of exogenous genes. Furthermore, unlike deep brain stimulation, a surgical electrical therapy that interfaces with neural tissue through implanted electrodes, noninvasive terahertz waves can affect cortical regions under the skull due to their high transmittance, as demonstrated in previous studies [6,7].

Since the vibrations and rotations of biomolecules predominantly occur in the high-frequency terahertz range, these bio-intervention studies focus on this high-frequency terahertz band on central-nervous-system-related behaviors [8]. The anterior cingulate cortex (ACC), a brain region located beneath the skull, is reported to be important for the regulation of pain and anxiety [9]. Our previous work demonstrates 36-THz high-frequency terahertz stimulation (HFTS), delivered through a pre-embedded cannula into the ACC, effectively alleviates the neuronal hyperactivities in the ACC and neuropathic pain [10]. A recent study from Song et al. [11] also showed that 34-THz stimulation alleviates inflammatory pain and pain-induced anxiety-like behaviors in mice by direct inhibition of hydrogen bonds bound between glutamate and its receptor in the ACC, using a similar implanted optic fiber illumination method. However, while pain-related anxiety represents a substantial factor, it is not the main cause of anxiety. Stress stemming from various aspects of life often serves as a more critical factor in the onset of anxiety. The ACC exhibits increased neural activity in both experimental animal studies and clinical neuroimaging data during stress-induced anxiety processing [12,13]. Meanwhile, the cellular mechanism for pain-induced anxiety, which is closely dependent on the establishment of pain, is quite different from the common anxiety induced by stress [14,15]. Thus, it is essential to explore whether a noninvasive application of HFTS can alleviate anxiety in stressed mice by acting on the neuronal activities of the ACC, potentially serving as clinical therapy.

In the present study, we first subjected mice to a 2-h acute restraint stress (ARS), a commonly used model for inducing anxiety, and then clustered the stressed mice into anxiety-susceptible and anxiety-resilient groups using the *K*-means algorithm, in order to mimic the clinical manifestation of anxiety in response to stress. An anxiety phenotype prediction classifier (APPC) was developed using the naïve Bayes (NB) algorithm, and anxiety-susceptible mice were selected to test the effect of transcranial HFTS delivered across the skull above the ACC. For the first time, we verify that stress-induced anxiety-like behavioral responses in ARS mice were markedly alleviated by noninvasive application of HFTS. This anxiolytic effect treatment expands the potential applications of HFTS in regulating diverse nervous system disorders.

## Results

### Anxiety-like behavioral responses were induced in mice subjected to ARS

**Fig. 1.**
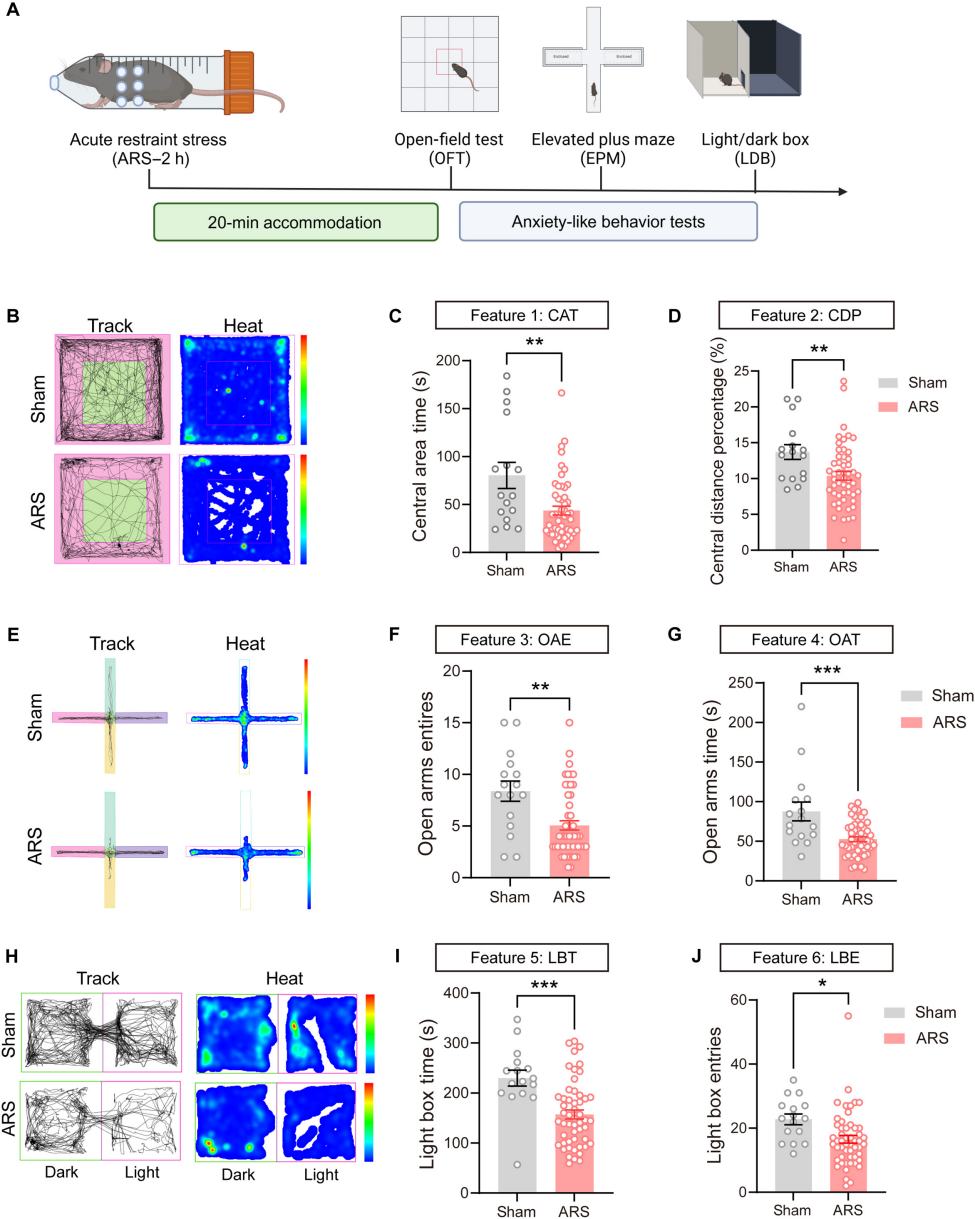
Acute stress induced anxiety-like behaviors in mice. (A) Schematic of anxiety-like behavior design. (B) Representative activity track maps and heat maps of the open-field test (OFT) from the indicated groups. (C) Summarized data showing a decrease in central area time (CAT) after acute restraint stress (ARS) induction (sham vs. ARS: *P* = 0.0015, *n*_sham_ = 16, *n*_ARS_ = 52, unpaired *t* test). (D) Summarized data showing decreased central distance percentage (CDP) after ARS induction (sham vs. ARS: *P* = 0.0089, *n*_sham_ = 16, *n*_ARS_ = 52, unpaired *t* test). (E) Representative activity track maps and heat maps of the elevated plus maze (EPM) from the indicated groups. (F) Summarized data showing decreased open arms entries (OAE) after ARS induction (sham vs. ARS: *P* = 0.0011, *n*_sham_ = 16, *n*_ARS_ = 52, unpaired *t* test). (G) Summarized data showing decreased open arms time (OAT) after ARS induction (sham vs. ARS: *P* = 0.0001, *n*_sham_ = 16, *n*_ARS_ = 52, unpaired *t* test). (H) Representative activity track maps and heat maps of the light/dark box (LDB) from the indicated groups. (I) Summarized data showing decreased light box time (LBT) after ARS induction (sham vs. ARS: *P* = 0.0002, *n*_sham_ = 16, *n*_ARS_ = 52, unpaired *t* test). (J) Summarized data showing decreased light box entries (LBE) after ARS induction (sham vs. ARS: *P* = 0.0106, *n*_sham_ = 16, *n*_ARS_ = 52, unpaired *t* test). *P < 0.05; **P < 0.01; ***P < 0.001; ****P < 0.0001.

We first tested behavioral indicators of anxiety in mice subjected to a 2-h ARS. A series of behavioral tests were conducted, beginning with the open-field test (OFT), followed by the elevated plus maze (EPM) test and culminating in the light/dark box (LDB) test. A 90-min interval was allowed between behavioral tests (Fig. 1A). In the OFT, ARS mice exhibited a significant decrease in central area time (CAT, *P* = 0.0015) and a lower central distance percentage (CDP, *P* = 0.0089) compared to the control mice (Fig. 1B to D). Moreover, the EPM test revealed that ARS mice had significantly fewer entries into the open arms (OAE, *P* = 0.0011) and spent less time in open arms (OAT, *P* = 0.0001) in comparison with the control mice (Fig. 1E to G). In the LDB test, the time spent in the light box (LBT, *P* = 0.0002) and entries into the light box (LBE, *P* = 0.0106) were decreased significantly compared to those of the control mice (Fig. 1H to J). Collectively, these experiments suggest that anxiety-like behaviors are induced in ARS mice.

### ARS enhanced the activity level of ACC pyramidal neurons

**Fig. 2.**
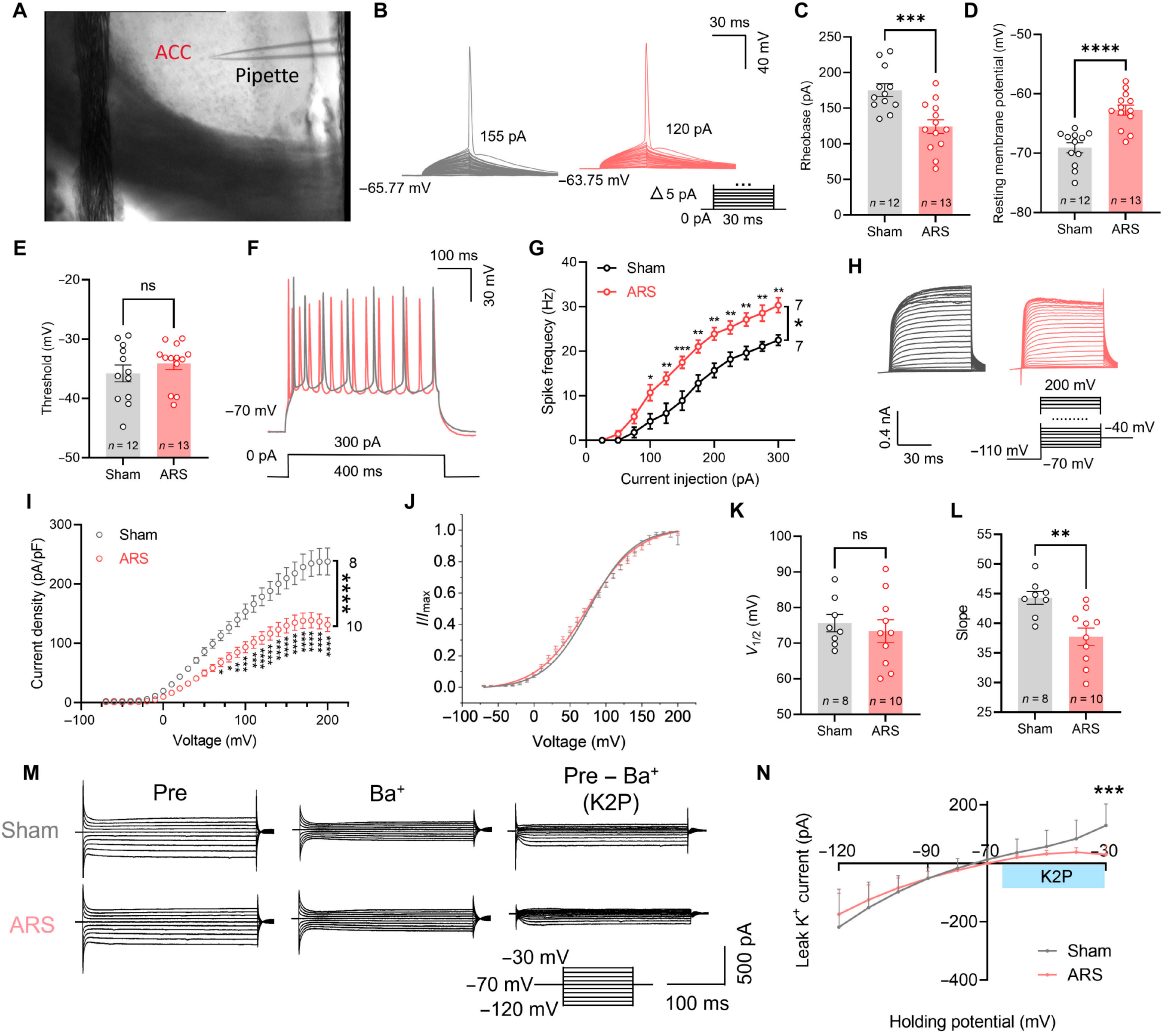
The impact of acute stress on the excitability and voltage-dependent potassium channels (Kv) currents of pyramidal neurons in the anterior cingulate cortex (ACC) of mice. (A) Anatomical location of the ACC region in mice with a pipette. (B to G) The impact of ARS on the excitability of pyramidal neurons. (B) Superimposed traces showing the single action potential (AP) evoked by a step current stimulation (inset) in different groups. (C) Bar graphs indicating a decrease in the rheobase of AP after ARS (sham vs. ARS: *P* = 0.0008 < 0.001, *n*_sham_ = 12, *n*_ARS_ = 13, unpaired *t* test). (D) Bar charts showing an increase in resting membrane potential after ARS (sham vs. ARS: *P* < 0.0001, *n*_sham_ = 12, *n*_ARS_ = 13, unpaired *t* test). ARS has no significant effect on the threshold (E) (sham vs. ARS: *P* = 0.3398 > 0.05, *n*_sham_ = 12, *n*_ARS_ = 13, unpaired *t* test). Representative traces (F) and line charts (G) showing changes in the evoked spikes of pyramidal neurons in different groups (sham vs. ARS: *F*_(11, 144)_ = 1.92, *P* = 0.0421 < 0.05, *n*_sham_ = 7, *n*_ARS_ = 7, 2-way analysis of variance [ANOVA] followed by post hoc comparison using Šídák’s multiple comparisons test). (H to L) The impact of ARS on the Kv current of pyramidal neurons. (H) Representative Kv currents evoked by a series of step voltages (inset) without (gray) or with ARS (pink). (I) *I*–*V* scatter plots constructed from the values of traces shown in (H) display a weaker Kv current with ARS (sham vs. ARS: *F*_(27, 448)_ = 6.113, *P* ≤ 0.0001, *n*_sham_ = 8, *n*_ARS_ = 10, 2-way ANOVA followed by post hoc comparison using Šídák’s multiple comparisons test). (J) The activation curves of the Kv currents in different groups. (K) The corresponding half-activation voltages of the activation curves (sham vs. ARS: *P* = 0.5963 > 0.05, *n*_sham_ = 8, *n*_ARS_ = 10, unpaired *t* test). (L) The corresponding slopes of the activation curves (sham vs. ARS: *P* = 0.0035 < 0.001, *n*_sham_ = 8, *n*_ARS_ = 10, unpaired *t* test). (M and N) The impact of ARS on the leak potassium channel (K2P) current of pyramidal neurons. (M) Representative K2P currents evoked by a series of step voltages (inset) without (gray) or with ARS (pink). (N) *I*–*V* scatter plots constructed from the values of traces shown in (M) display a weaker K2P current with ARS (sham vs. ARS: *F*_(9, 170)_ = 2.773, *P* = 0.0047 < 0.01, *n*_sham_ = 9, *n*_ARS_ = 10, 2-way ANOVA followed by post hoc comparison using Šídák’s multiple comparisons test). *P < 0.05; **P < 0.01; ***P < 0.001; ****P < 0.0001; ns, *P* > 0.05.

To examine how acute stress modulates neuronal electrophysiological properties in the ACC—a region critically linked to anxiety [12]—we performed whole-cell patch-clamp recordings on layer V pyramidal (PYR) neurons in acute brain slices from mice subjected to ARS (Fig. 2A). We applied a 30-ms suprathreshold depolarizing current pulse to PYR neurons to elicit a single action potential (AP; Fig. 2B). Analysis of PYR membrane properties revealed a reduced rheobase (*P* = 0.0008) and an elevated resting membrane potential (RMP; *P* < 0.0001) in ARS mice relative to those of sham controls (Fig. 2C to E). Th Input–output curves of evoked APs were then induced by a 400-ms suprathreshold current pulse (Fig. 2F and G). Our results indicated a notable rise in the number of spikes observed in ARS mice (*P* = 0.0421, Table S1). These findings indicate that ARS enhances the excitability of PYR neurons. Meanwhile, these results strongly suggest that the voltage-gated potassium channels (Kv), which regulate the firing of AP [16], and leak potassium channels (K2P), which regulate the RMP [17], are involved in the hyperactivity of PYR^ACC^ neurons in ARS mice.

We thus tested whether the Kv and K2P currents were changed in ARS mice. To isolate the Kv currents, we employed a protocol involving a sequence of 100-ms test pulses ranging from −70 to +200 mV, each preceded by a 20-ms prepulse to −110 mV. This was conducted in the presence of tetrodotoxin (TTX; 1 μM) and CdCl_2_ (100 μM) to block voltage-gated Na^+^ and Ca^2+^ currents (Fig. 2H). The findings revealed that ARS caused a notable reduction in Kv current density (*P* < 0.0001) and a decreased slope of the activation curve (*P* = 0.0035), while the half-activation voltage remained unchanged (Fig. 2I to L and Table S2). Furthermore, we tested the possibly changed K2P current. To isolate BaCl_2_-sensitive components, test pulses (400-ms duration, −70 to −30 mV) were applied after a 50-ms conditioning period at −70 mV, with all recordings performed under 4 mM BaCl_2_ (Fig. 2M). These results showed that ARS induced a significant decrease in currents with the holding potential from −50 to −30 mV (*P* = 0.0047, Fig. 2N and Table S3), without affecting the currents with a holding potential lower than −70 mV, suggesting that ARS decreases the K2P currents but not the inwardly rectifying K^+^ (Kir) currents. The collective findings indicate that ARS enhances the PYR^ACC^ neuronal activity through suppression of their Kv and K2P currents.

### Anxiety phenotype clustering using the *K*-means algorithm in ARS mice

**Fig. 3.**
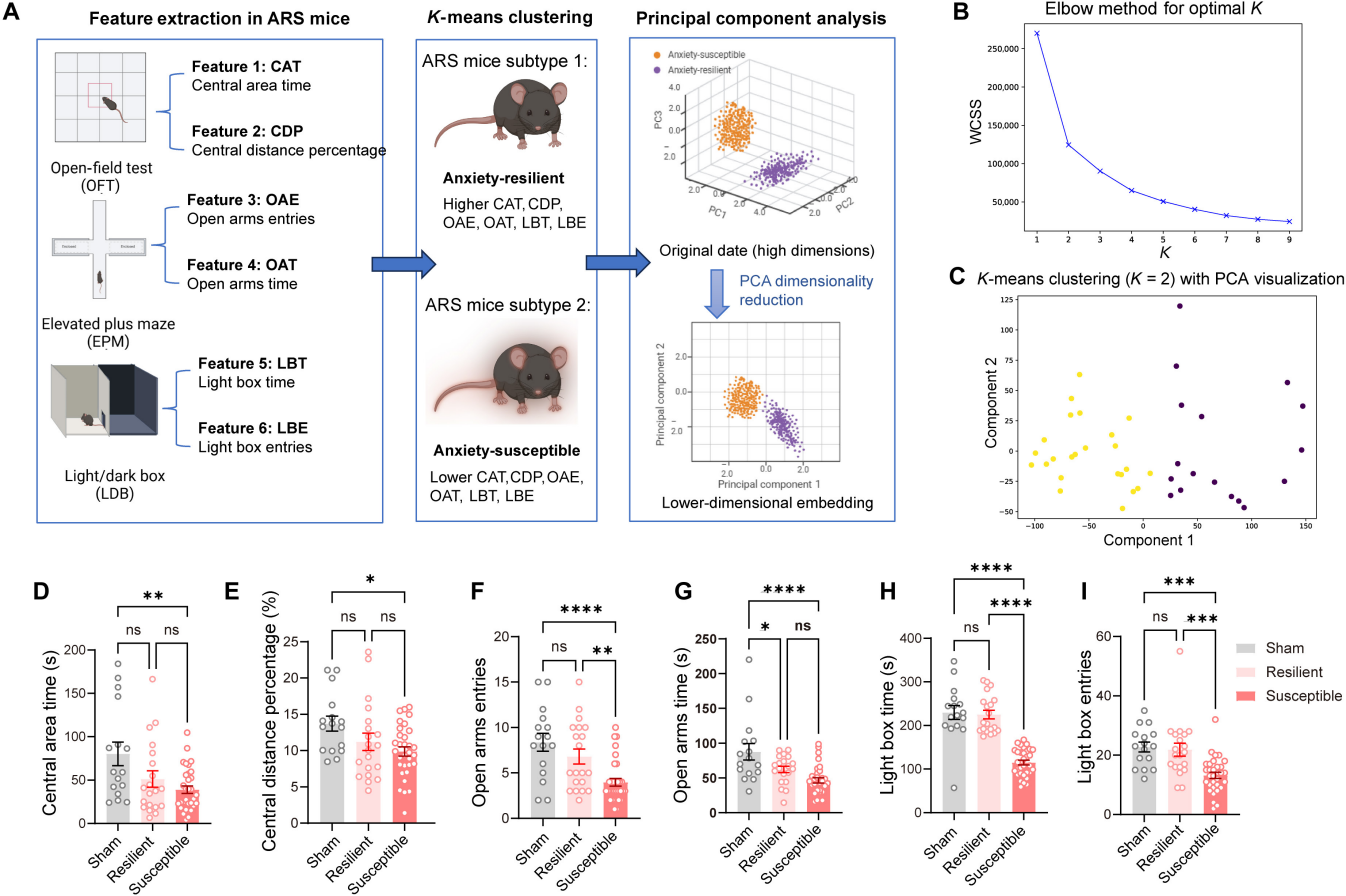
Anxiety phenotype clustering by using the *K*-means algorithm based on 6 features in ARS mice. (A) The process of anxiety phenotype clustering involved feature extraction, *K*-means clustering, and visualization through principal component analysis (PCA). (B) The optimal number of clusters, *K* = 2, was automatically determined using the elbow method, allowing the anxiety phenotype to be divided into 2 distinct subtypes. WCSS, within-cluster sum of squares. (C) PCA visualization transformed the high-dimensional original data into a 2-dimensional space, where the 2 subtypes were clearly distinguishable. (D to I) Based on the *K*-means clustering results, the behavioral performance of 52 ARS mice across 6 features was classified into anxiety-resilient and anxiety-susceptible categories. (D) Histograms showing the statistical comparison of central area time in each group (sham vs. susceptible: *P* = 0.0025). (E) Histograms showing the statistical comparison of central distance percentage in each group (sham vs. susceptible: *P* = 0.0136). (F) Histograms showing the statistical comparison of open arms entries in each group (sham vs. susceptible: *P* < 0.0001; resilient vs. susceptible, *P* = 0.0079). (G) Histograms showing the statistical comparison of open arms time in each group (sham vs. resilient: *P* = 0.0330; sham vs. susceptible: *P* < 0.0001). (H) Histograms showing the statistical comparison of light box time in each group (sham vs. susceptible: *P* < 0.0001; resilient vs. susceptible, *P* < 0.0001.). (I) Histograms showing the statistical comparison of light box entries in each group (sham vs. susceptible: *P* < 0.0001; resilient vs. susceptible, *P* = 0.0079.). *n*_sham_ = 16, *n*_resilient_ = 20, and *n*_susceptible_ = 32. One-way ANOVA followed by post hoc comparison using Tukey’s multiple comparisons test was used. *P < 0.05; **P < 0.01; ***P < 0.001; ****P < 0.0001; ns, *P* > 0.05.

We proposed that some of the ARS mice may be resilient to anxiety, since their behavioral data were similar to those in control groups (Fig. 1). To exclude the anxiety-resilient mice in the ARS group, clustering analysis based on the *K*-means algorithm was utilized as demonstrated in prior research from our group [18]. CAT and CDP in OFT, OAE and OAT in the EPM maze test, and LBT and LBE in the LDB test were used as 6 features in clustering analysis, to provide an unbiased assessment of anxiety status in each individual mouse (Fig. 3A). The optimal number of clusters for *K*-means is determined using the elbow method, with the “elbow” point of the highest curvature occurring at *K* = 2 (Fig. 3B). The elbow method can automatically determine the optimal number of clusters, resulting in improved clustering. Following this, the dataset is visualized using principal component analysis, which clearly shows the 2 distinct clusters (Fig. 3C). The mice in one cluster were labeled as anxiety-resilient mice, which show higher values of the 6 features, and the mice in the other cluster were named as anxiety-susceptible mice, which show lower values of the 6 features when compared with the sham group (Fig. 3D). Among the features, LBT/LBE in the LDB test (*P* < 0.0001) showed the most significant differences between the anxiety-resilient group and the anxiety-susceptible group, indicating that these features have better discrimination.

### An APPC developed using the NB algorithm

**Fig. 4.**
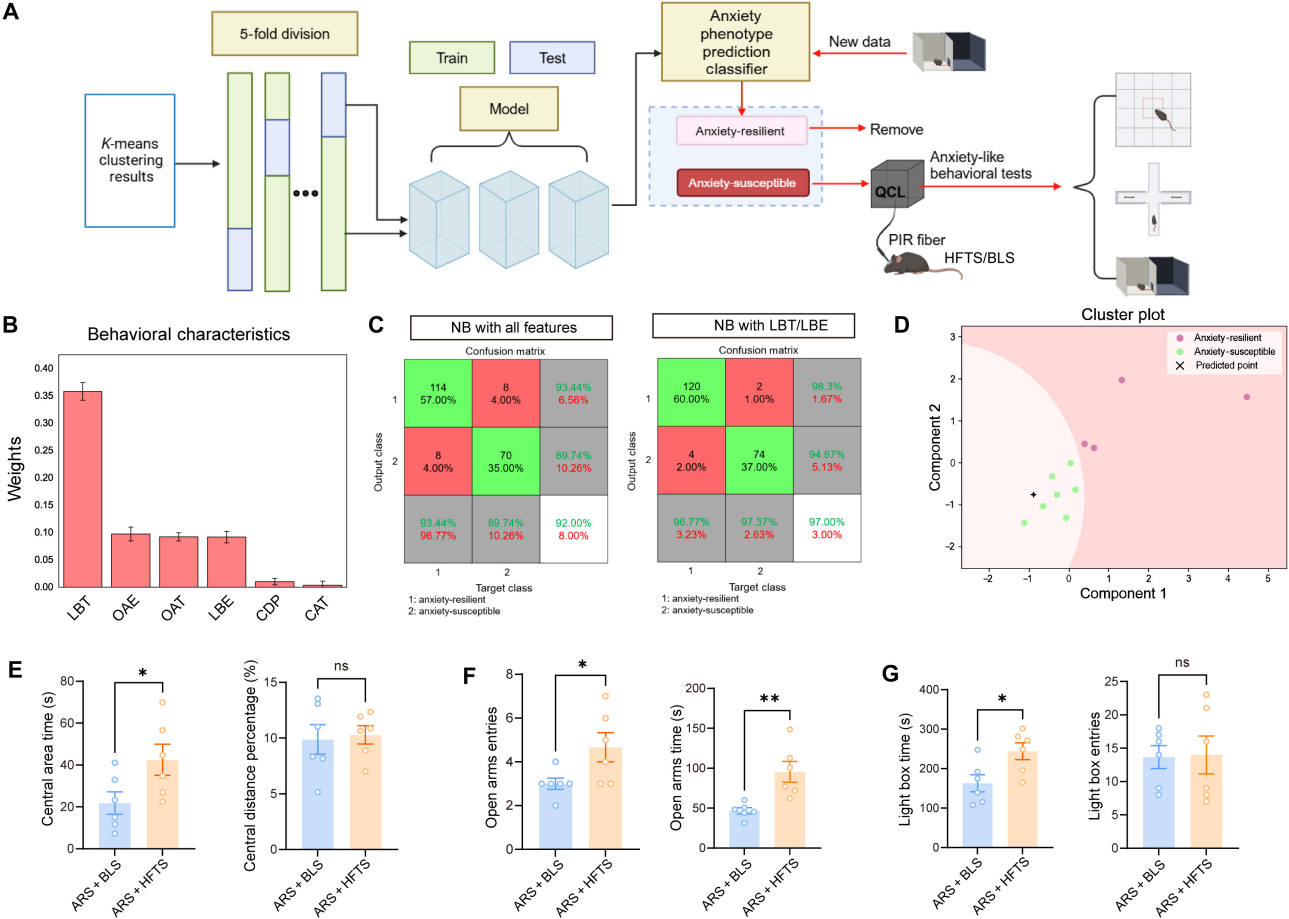
Noninvasive high-frequency terahertz stimulation (HFTS) alleviated anxiety in anxiety-susceptible mice predicted by naïve Bayes (NB) classifier based on 2 features of LDB. (A) The experimental framework consisted of data preprocessing, 5-fold division (5D) feature establishment, feature selection, and model training, and then new experimental data were input to determine the anxiety phenotype of mice, and anxiety-susceptible mice were subjected to HFTS/blue light stimulation (BLS) for behavioral tests. (B) The mean weights of different features obtained with ReliefF. (C) Confusion matrixes of NB with all features (left) and NB with LBT/LBE (right). (D) Graph of the mouse anxiety phenotype classifier and distribution of testing dataset and new data prediction. (E) Histograms showing the behavioral changes in OFT after noninvasive HFTS application. Left: summarized data showing increased CAT after HFTS/BLS in ARS mice (ARS + BLS vs. ARS + HFTS: *P* = 0.0476). Right: summarized data showing CDP after HFTS/BLS in ARS mice (ARS + BLS vs. ARS + HFTS: *P* = 0.7996). (F) Histograms showing the behavioral changes in EPM after noninvasive HFTS application. Left: summarized data showing OAE after HFTS/BLS in ARS mice (ARS + BLS vs. ARS + HFTS: *P* = 0.0420). Right: summarized data showing OAT after HFTS/BLS in ARS mice (ARS + BLS vs. ARS + HFTS: *P* = 0.0051). (G) Histograms showing the behavioral changes in LDB after noninvasive HFTS application. Left: summarized data showing LBT after HFTS/BLS in ARS mice (ARS + BLS vs. ARS + HFTS: *P* = 0.0241). Right: summarized data showing LBE after HFTS/BLS in ARS mice (sham vs. ARS: *P* = 0.9223), *n*_ARS+BLS_ = 6, *n*_ARS+HFTS_ = 6, unpaired *t* test. *P < 0.05; **P < 0.01; ns, *P* > 0.05. QCL, quantum cascade laser; PIR, polycrystalline infrared.

Animals should not undergo the same anxiety apparatus twice, as repeated exposure may result in leaking of motivation to explore the open or lit space, thus leading to a gradual increase in avoidance behavior and a decision to remain in the safer areas of the maze [19–21]. To minimize the number of times that each animal underwent the same behavioral experiment, we developed a classifier capable of predicting anxiety phenotypes based on the most dominant behavioral test (Fig. 4A). To identify this predominant test, we calculated the contribution score of each feature to the classification outcome and selected the most discriminative one by using the ReliefF algorithm [22]. The weight of the LBT was approximately 0.35, and another feature of the LDB, LBE, ranked fourth among the behavioral characteristic weights (Fig. 4B). Consequently, we selected LBT and LBE as the 2 features in the LDB test to predict the anxiety phenotype in each mouse. To address numerical differences that could impact model training, we standardized the features by computing their mean and variance for data preprocessing. In the present study, 5-fold cross-validation was used to test the stability and generalization of the model. In each round of validation, the dataset was randomly divided into training (80%) and test (20%) sets. The results from the 5 rounds were averaged to obtain the final performance metrics. To establish robustness, we leveraged 2 prominent machine learning algorithms, random forest (RF) and NB, for comparative performance assessment. Additionally, to validate the effectiveness of the most discriminative features selected by ReliefF, we trained the APPC model using both all available features and the subset of LBT/LBE features. The classification results of various models and classifiers were assessed using a confusion matrix, and the best-performing classifier was chosen as the standard for future experiments. The NB classifier based on LBT and LBE reached an accuracy of 97%, surpassing that of the NB classifier utilizing all features (Fig. 4C). Furthermore, the accuracy of the NB classifier with 2 features was higher than that of RF with 2 features (Fig. S1B). The distribution of the testing sets is presented based on the APPC utilizing these 2 LDB features (Fig. 4D).

### Noninvasive HFTS alleviated anxiety-like behaviors in anxiety-susceptible mice

Using this APPC, we identified anxiety-susceptible mice and subjected them to tests with the application of either HFTS or control 473-nm blue light stimulation (BLS), while anxiety-resilient mice were excluded from the following study (Fig. 4A). The fiber was secured using a double-end fixture, ensuring that the fiber tip made contact with the skull at the specific thinned site above the right side of ACC (Fig. S2A). Representative trajectories of the anxiety-like behaviors are shown in Fig. S2B to D. Histograms demonstrate a marked enhancement in CAT in OFT (*P* = 0.0476), OAE, and OAT in EPM (*P* = 0.0051, *P* = 0.0420), and LBT in LDB (*P* = 0.0241) (Fig. 4E to G). These findings indicate that HFTS effectively alleviated anxiety-like behaviors in anxiety-susceptible mice compared to those subjected to BLS.

### Noninvasive HFTS inhibited neuronal activity in the ACC

**Fig. 5.**
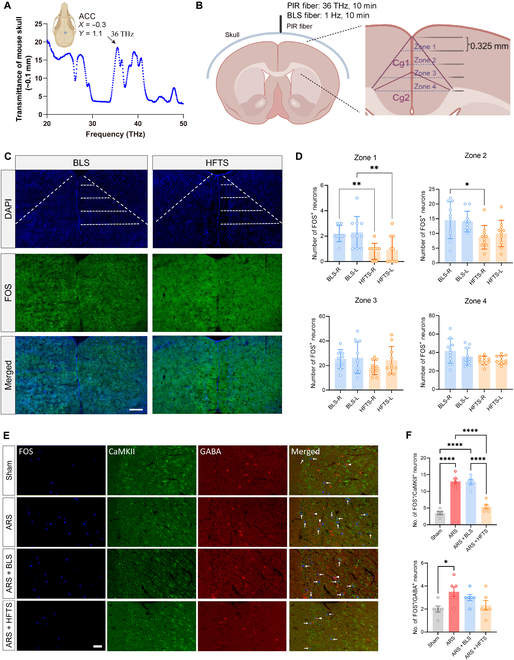
Noninvasive application of HFTS inhibited neuronal activities at the ACC in ARS mice. (A) Transmittance spectra of the skull above the ACC brain region. (B) Left image: schematic diagram showing the noninvasive approach design of HFTS delivery to the mouse brain at a thinned skull above the ACC. Right image: an enlarged picture showing the zoning of the ACC for counting statistics. (C) Zoning of confocal images of FOS and 4′,6-diamidino-2-phenylindole (DAPI) in the ACC near the target spot, 2 h after noninvasive application of HFTS/BLS. BLS, blue light stimulation. White dashed line: outlines the 4 zones of FOS neurons. The scale bar represents 300 μm. (D) Histograms of the numbers of FOS^+^ neurons in each zone; data points are pooled from 10 slices of 10 mice in each group. Zone 1: BLS-R vs. HFTS-R: *P* = 0.0085; BLS-L vs. HFTS-L: *P* = 0.0085. Zone 2: BLS-R vs. HFTS-R: *P* = 0.0350. One-way ANOVA followed by Šídák’s multiple comparisons test. *P < 0.05; **P < 0.01. (E) Immunofluorescence staining for FOS/calcium/calmodulin-dependent protein kinase II (CaMKII)/γ-aminobutyric acid (GABA) in zone 2 of the ACC from the sham, ARS, ARS + BLS, and ARS + HFTS groups. The scale bar represents 50 μm. (F) Histograms showing the numbers of FOS^+^/CaMKII^+^ and FOS^+^/GABA^+^ neurons in zone 2 of the ACC. One-way ANOVA followed by Šídák’s multiple comparisons test. *P < 0.05; ****P < 0.0001.

A previous study showed 53-Hz HFTS through an opened skull or across a thinned skull activates neurons in the targeted auditory cortex [7]. To determine if the 36-THz HFTS used in the present study can be delivered to the ACC noninvasively, we assessed the transmittance spectra of the skull by using a Fourier transform infrared spectrometer. It was found that the skull sample had a transmittance of 20% (Fig. 5A) at 36 THz. These results establish a foundation for noninvasive terahertz modulation of ACC neuronal activity beneath the skull. Consequently, we delivered HFTS by positioning optic fibers on the skull overlying the right side (0.3 mm lateral) of ACC (ACC^R^). The skull was slightly thinned for about 0.1 mm to form a shallow well to help fix the head of the optic fiber with a double-end fixture (Fig. 5B). The extent of HFTS’s influence range within the ACC was further explored through FOS immunofluorescent staining [23]. The ACC was divided into 4 zones along the longitudinal axis, each zone being 0.325 mm deep, to facilitate the counting of the number of FOS-immunoreactive (FOS^+^) neurons. We found that HFTS markedly suppressed the number of FOS^+^ neurons, especially at zone 1 and zone 2, compared to that in the ACC^R^ of mice by BLS (1 Hz, 10 min). However, zones 3 and 4 did not exhibit this difference, suggesting that the influence depth of HFTS may not exceed 0.65 mm. We also noticed a decreased Fos expression in zone 1 and zone 2 of the ACC^L^ (Fig. 5D). Considering that the terahertz light emitted from the optical fiber should spread outward in a conical shape, we believe that the terahertz light also diffused to some extent into the superficial regions on the ACC^L^, thereby inhibiting the activity of neurons inside.

After performing triple immunofluorescence staining for FOS/calcium/calmodulin-dependent protein kinase II (CaMKII)/γ-aminobutyric acid (GABA) in the ACC of each group of mice, we observed that the number of CaMKII-immunoreactive (CaMKII^+^) pyramidal neurons expressing FOS protein in zone 2 of ACC was significantly reduced in the ARS + HFTS group compared to those in the ARS + BLS and ARS groups, while the number of FOS-expressing GABA-immunoreactive (GABA^+^) interneurons in the ARS + HFTS group showed no statistical difference when compared with that in the ARS + BLS or ARS group (Fig. 5E and F). These results indicate that HFTS primarily exerts its anxiolytic effect by reducing the activity of pyramidal neurons in the ACC.

### HFTS inhibited the excitability of clustered units in the ACC in vivo

**Fig. 6.**
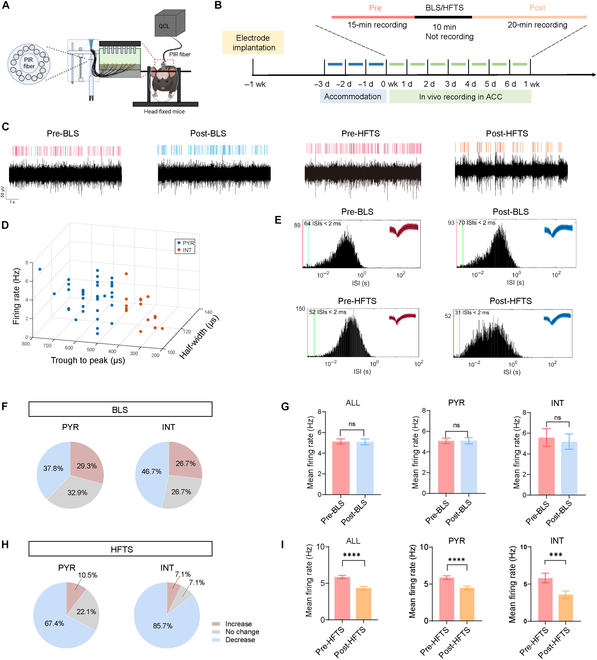
HFTS effectively suppressed the spiking activity of pyramidal neurons in the ACC of head-fixed mice. (A) Schematic diagram of in vivo single-unit recording by a custom-designed 16-channel electrode in head-fixed awake mice. (B) The timeline and procedures for administering HFTS and BLS to head-fixed mice. (C) Representative recording signals of ACC neurons in ARS mice. The red lines indicate the spikes before application of BLS and HFTS. The blue lines indicate the spikes after BLS application. The orange lines indicate the spikes after HFTS application. (D) The clustered units are classified into pyramidal neurons (PYR) and interneurons (INT) using a *K*-means cluster-separation algorithm based on their electrophysiological properties. (E) Histograms of the interspike intervals (ISIs) from the spikes of a PYR and an INT in pre- and post-BLS and HFTS recording period. Insets at the top right corner show the waveforms of the detected single unit. (F) Pie charts summarizing the changes in the firing rate of PYR and INT in the ARS + BLS group. Pre- vs. post-BLS, Wilcoxon rank-sum test. (G) The mean firing rate of all recorded units (ALL), PYR^ACC^ neurons (PYR), and INT^ACC^ neurons (INT) in the ARS + BLS group before and after BLS. ALL (*P* = 0.4096, *n* = 93, Wilcoxon matched-paired signed rank test), PYR (*P* = 0.5437, *n* = 82, Wilcoxon matched-paired signed rank test), and INT (*P* = 0.5195, *n* = 11, Wilcoxon matched-paired signed rank test). (H) Pie charts summarizing the changes in the firing rate of PYR and INT in the ARS + HFTS group. Pre- vs. post-HFTS, Wilcoxon rank-sum test. (I) The mean firing rate of all recorded units (ALL), PYR^ACC^ neurons (PYR), and INT^ACC^ neurons (INT) in the ARS + HFTS group before and after HFTS. ALL (*P* < 0.0001, *n* = 100, Wilcoxon matched-paired signed rank test), PYR (*P* < 0.0001, *n* = 86, Wilcoxon matched-paired signed rank test), and INT (*P* = 0.0004, *n* = 14, Wilcoxon matched-paired signed rank test). ***P < 0.001; ****P < 0.0001; ns, *P* > 0.05.

Subsequently, we evaluated the effects of HFTS on the activity of diverse neuronal subtypes in awake, head-restrained mice. A week before recordings, custom-designed 16-channel electrodes were surgically placed in the ACC to enable precise neural activity monitoring (Fig. 6A). Neuronal activities were compared before and after stimulation with either 1-Hz BLS or 36-THz HFTS (Fig. 6B; see Methods for stimulation parameters). Our data demonstrated that HFTS application in the ARS + HFTS group resulted in a significant suppression of spontaneous firing activity in ACC neurons (Fig. 6C). The cell-type classification (PYR^ACC^ vs. INT^ACC^) based on the trough-to-peak duration, firing rate, and half-width (Fig. 6D) was essential for isolating HFTS effects specific to pyramidal neurons [24]. To validate single-unit recording stability, we systematically compared the interspike interval distributions and waveform parameters of clustered units across all channels before versus after BLS/HFTS interventions (Fig. 6E). In the ARS + BLS group, 37.8% of PYR^ACC^ neurons showed a decline in firing rate, while 29.3% showed an increase, and 32.9% remained unchanged (Fig. 6F). The mean firing rate of PYR^ACC^ and INT^ACC^ in ARS + BLS mice showed no obvious change by BLS (Fig. 6G). Consistently, in the ARS + HFTS group, 46.7% of PYR^ACC^ neurons showed decreased activity, 26.7% showed increased activity, and 26.7% remained unchanged (Fig. 6H). The mean firing rate of all the clustered units, PYR^ACC^ neurons, and INT^ACC^ neurons was significantly suppressed by HFTS application in the ARS + HFTS group (Fig. 6I).

Since both the PYR^ACC^ and INT^ACC^ neurons were inhibited by HFTS, we further checked the excitation/inhibition (E/I) ratio of the PYR^ACC^ neurons to clarify the net effect of HFTS. PYR^ACC^ and INT^ACC^ were distinguished based on their distinct spiking patterns and membrane properties, as well as the immunofluorescence staining characteristics observed after the recording (Fig. S3A and B). Our results demonstrated that HFTS significantly suppressed presynaptic glutamate and GABA release. This was evidenced by an increase in the paired pulse ratio (PPR) of the electrically evoked excitatory postsynaptic current (eEPSC) and electrically evoked excitatory postsynaptic current inhibitory postsynaptic current (eIPSC), along with a decrease in the frequency of the miniature excitatory postsynaptic current (mEPSC) and miniature inhibitory postsynaptic current (mIPSC). Simultaneously, HFTS reduced postsynaptic responses, as indicated by a decrease in the amplitudes of mEPSCs and mIPSCs (Fig. S3C, D, and H to L and Table S4). Notably, both the amplitude ratio and frequency ratio of mEPSC/mIPSC decreased (Fig. S3M and Table S4), suggesting that the overall effect of HFTS is to reduce the excitatory synaptic inputs on PYR^ACC^ neurons. Consistent with this, although HFTS reduced the amplitudes of both eEPSCs and eIPSCs, the eEPSC/eIPSC ratio decreased significantly (Fig. S3E to G and Table S4), indicating that the net effect of HFTS on PYR^ACC^ neurons was to reduce their excitability.

The results above indicate that HFTS should directly or indirectly modulate the function of α-amino-3-hydroxy-5-methyl-4-isoxazolepropionic acid receptors (AMPARs) and γ-aminobutyric acid type A receptors (GABA_A_Rs) on PYR^ACC^ neurons. Therefore, we designed further experiments to test the possible correlations between K^+^ channels and the function of AMPARs or GABA_A_Rs after HFTS. We found that in the presence of Kv and K2P channel blockers (4-aminopyridine [4-AP] and BaCl_2_), HFTS no longer affected the frequency and amplitude of mEPSCs and mIPSCs and the amplitude ratio and frequency ratio of mEPSC/mIPSC (Fig. S3H to M and Table S4).

### HFTS inhibited ACC pyramidal neurons in ARS mice by increasing voltage-gated K^+^ currents

**Fig. 7.**
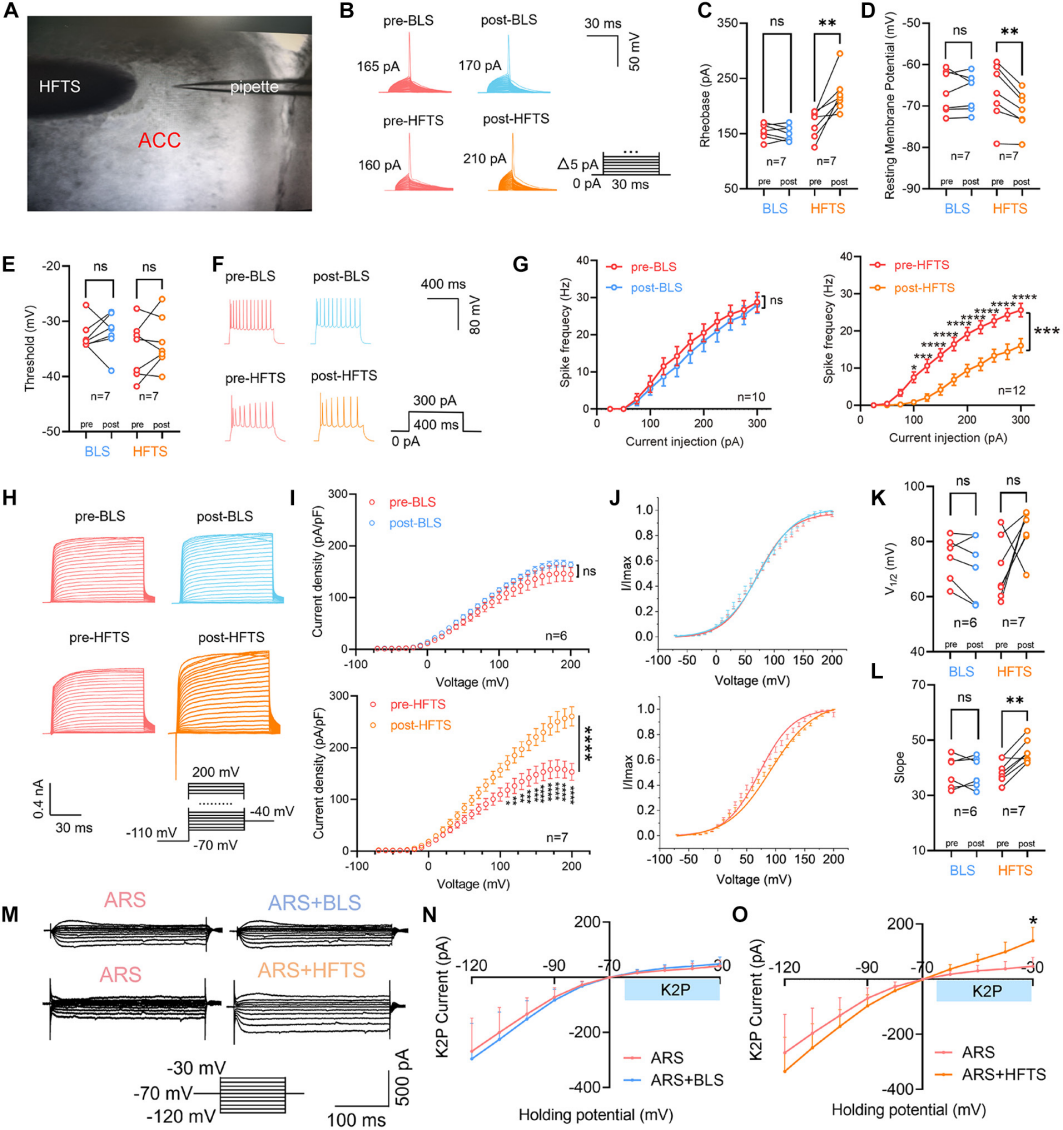
HFTS reduces the APs’ firing rate and enhances the voltage-dependent potassium channel (Kv) currents of pyramidal neurons in ARS mice in vitro. (A) Anatomical location of the ACC region in mice with an HFTS fiber and a pipette. (B) Superimposed traces showing the single AP evoked by step current stimulation (inset) in different groups. (C) Line charts indicating an increase in the rheobase of AP after the administration of the HFTS but not BLS in ARS mice (pre-BLS vs. post-BLS: *P* = 0.3758 > 0.05, *n* = 7, paired *t* test; pre-HFTS vs. post-HFTS: *P* = 0.0076 < 0.01, *n* = 7, paired *t* test). (D) Line charts showing a decrease in resting membrane potential after the administration of HFTS but not BLS in ARS mice (pre-BLS vs. post-BLS: *P* = 0.9151 > 0.05, *n* = 7, paired *t* test; pre-HFTS vs. post-HFTS: *P* = 0.004 < 0.01, *n* = 7, paired *t* test). (E) Both HFTS and the BLS have no significant effect on the threshold (pre-BLS vs. post-BLS: *P* = 0.6729 > 0.05, *n* = 7, paired *t* test; pre-HFTS vs. post-HFTS: *P* = 0.3839 > 0.05, *n* = 7, paired *t* test). Representative traces (F) and line charts (G) showing changes in the evoked spikes of pyramidal neurons in different groups. HFTS can decrease the frequency of spikes (G, right) in the pyramidal neurons of ARS mice (pre-HFTS vs. post-HFTS: *F*_(11, 264)_ = 3.551, *P* = 0.0001 < 0.001, *n* = 12, 2-way ANOVA followed by post hoc comparison using Šídák’s multiple comparisons test), while BLS (G, left) cannot (pre-BLS vs. post-BLS: *F*_(11, 216)_ = 0.1136, *P* = 0.9998 > 0.05, *n* = 10, 2-way ANOVA followed by post hoc comparison using Šídák’s multiple comparisons test). (H to L) The impact of HFTS or BLS on the Kv current of pyramidal neurons. (H) Representative Kv currents evoked by a series of step voltages (inset) in different groups. (I) *I*–*V* scatter plots constructed from the values of traces shown in (H) display a stronger Kv current with HFTS (down) but not BLS (up) (pre-BLS vs. post-BLS: *F*_(27, 280)_ = 0.4268, *P* = 0.9951 > 0.05, *n* = 6; pre-HFTS vs. post-HFTS: *F*_(27, 336)_ = 4.688, *P* < 0.0001, *n* = 7, 2-way ANOVA followed by post hoc comparison using Šídák’s multiple comparisons test). (J) The activation curves of the Kv currents in different groups. The corresponding half-activation voltages (K) of the activation curves (pre-BLS vs. post-BLS: *P* = 0.1713 > 0.05, *n* = 6; pre-HFTS vs. post-HFTS: *P* = 0.0631 > 0.05, *n* = 7, paired *t* test). The corresponding slopes (L) of the activation curves (pre-BLS vs. post-BLS: *P* = 0.9934 > 0.05, *n* = 6; pre-HFTS vs. post-HFTS: *P* = 0.0018 < 0.05, *n* = 7, paired *t* test). (M to O) The impact of HFTS and BLS on the K2P current of pyramidal neurons in ARS mice. (M) Representative K2P currents evoked by a series of step voltages with HFTS or BLS. (N) There are no differences in *I*–*V* scatter plots before and after BLS (sham vs. ARS: *F*_(9, 160)_ = 2.767, *P* = 0.9802 > 0.05, *n*_ARS_ = 9, *n*_ARS+BLS_ = 9, 2-way ANOVA followed by post hoc comparison using Šídák’s multiple comparisons test). (O) *I*–*V* scatter plots constructed from the values of traces shown in (M) display a stronger K2P current with HFTS (ARS vs. ARS + HFTS: *F*_(9, 120)_ = 2.696, *P* = 0.0068 < 0.01, *n*_ARS_ = 7, *n*_ARS+HFTS_ = 7, 2-way ANOVA followed by post hoc comparison using Šídák’s multiple comparisons test). *P < 0.05; **P < 0.01; ***P < 0.001; ****P < 0.0001; ns, *P* > 0.05.

In the final phase of our study, we examined how the characteristic resonance frequency of terahertz photons modulates the activity of PYR^ACC^ neurons in ARS mice (Fig. 7A). Through whole-cell current-clamp recordings, we analyzed changes in individual AP parameters following BLS/HFTS application compared to baseline conditions. HFTS application significantly elevated the rheobase (*P* = 0.0076) and reduced the RMP (*P* = 0.004), whereas BLS demonstrated no statistically significant effects (Fig. 7B to D). Furthermore, HFTS application caused a significant reduction in spike frequency in ARS mice (*P* = 0.0001), whereas BLS showed no such effect (Fig. 7F and G and Tables S5 and S6). These results suggest that HFTS but not BLS reduces the excitability of PYR neurons. Moreover, the impacts of HFTS and BLS on Kv currents were also investigated to explore the electrophysiology mechanism (Fig. 7H). The results showed a significant increase in Kv currents (*P* < 0.0001) and an increased slope of the activation curve (*P* = 0.0018) with the application of HFTS but not BLS (Fig. 7I to K and Tables S7 and S8). There are no differences in half-activation voltage of the activation curve before and after HFTS or BLS. Finally, the impact of ARS on K2P currents was detected (Fig. 7M and O), and we found a notable rise in K2P currents after the application of HFTS (*P* = 0.0068, Fig. 2N and Table S9) but not BLS (Fig. 7O and Table S10). Collectively, these results demonstrate that HFTS modulates PYR^ACC^ neuronal activity in ARS mice via selective regulation of Kv channels and K2P currents. Specifically, HFTS suppresses spike firing in ACC neurons, whereas BLS lacks comparable efficacy.

## Discussion

In this study, we revealed that HFTS acted as a noninvasive therapy to alleviate anxiety in mice susceptible to ARS, by targeting the ACC. This finding holds promise for the future medical application of terahertz waves. Additionally, we innovatively developed an APPC utilizing the NB algorithm, which accurately categorizes mice based on just 2 features from the LDB test. The APPC provides a reliable method for evaluating the impact of therapeutic drugs or physical factors on anxiety, eliminating the bias that arises from mice undergoing the same anxiety-like behavioral tests multiple times. Complementing these findings, our in vivo and in vitro electrophysiology analyses uncovered the mechanistic basis for HFTS-induced anxiolysis, demonstrating its ability to suppress PYR^ACC^ neuron excitability by enhancing both voltage-gated and leak potassium conductances.

Normative anxiety, an adaptive response caused by acute stress, represents a transient emotional state arising from perceived future threats. When anxiety becomes excessive and persistent with impairments in daily functioning, it is diagnosed as an anxiety disorder [25,26]. In mice, short-term anxiety induced by ARS has been confirmed to last up to 12 h and gradually dissipates upon removal of the stress stimuli [14,27]. Consequently, all experimental procedures were rigorously conducted within this 12-h window. Behavioral analyses revealed consistent, statistically significant differences across the 6 selected features when comparing sham and ARS mice. Notably, the interindividual variations observed in mice subjected to ARS mirror the clinical manifestation of anxiety responses to stress. This aligns with our previous finding that approximately two-thirds (67.9%) of mice with chronic neuropathic pain developed depressive symptoms [18]. Therefore, it is essential to identify all anxiety-susceptible mice for further investigation to validate the reliability of findings related to HFTS treatment effectiveness.

Anxiety phenotypes were clustered automatically into 2 subtypes using the *K*-means algorithm, for the optimal *K* value is 2 as determined by the elbow method. According to the clustered results, only 61.5% of the ARS mice were susceptible to anxiety. The reliability of the commonly used anxiety-related behavioral evaluations, namely, the OFT, EPM test, and LDB test, has been questioned in many studies due to the unequivocal measures, poor replicability, and a trade-off between innate curiosity and self-preservation in animals [21,28,29]. APPC predicts anxiety based on clear standards derived from calculations and machine learning, thus eliminating the need for multiple measures in behavioral tests when evaluating the anxiolytic effects of therapies. However, although the model proposed in this study exhibits a certain level of reliability, there are limitations that cannot be overlooked. Due to ethical considerations regarding animal use, it is challenging to significantly increase the sample size. Despite employing the bootstrap sampling method to expand the modeling training samples, there remains an urgent need for further research to expand the sample size and enhance the generalization capability of the classification model.

The mice susceptible to anxiety were classified using the APPC method and subsequently underwent HFTS application. The primary biomedical applications of terahertz waves focus on spectroscopic instruments and terahertz imaging systems [30,31]. While studies investigating the biological impacts of terahertz radiation are growing, the field remains underdeveloped. Several studies, including our previous research, have highlighted the advantage of terahertz technology: it has the capability to directly modulate neuronal firing activity, thereby affecting behavior and even pathological conditions, without the need for a transgene encoding photosensitive protein [7,10]. A recent study demonstrates that terahertz waves relieve pain-induced anxiety delivered by a fiber into the pre-embedded cannula [11]. However, the effect of terahertz waves on stress-induced anxiety, the main cause of anxiety, has never been investigated until now. We presented, for the first time, clear evidence from behavioral tests demonstrating the anxiety-relieving effect of HFTS in mice susceptible to anxiety. These mice were initially exposed to the LDB to elicit an APPC response and then underwent a series of experimental tests, including a repeated LDB test. Typically, in anxiety-like behavioral tests, mice exhibit a preference for remaining in safe areas when re-exposed to the same apparatus. However, our findings revealed a surprising increase in the time spent in the light box during the post-HFTS LDB test compared to that in the pre-HFTS test (Fig. S2D). This result further confirms the anxiolytic efficacy of noninvasive HFTS in anxiety-susceptible mice, providing the foundation for the clinical transcranial HFTS application for central nervous system disorders. Corticosterone measurements (hypothalamic–pituitary–adrenal axis activation), inflammatory markers (IL-6 and TNF-α), and their correlation with behavioral changes could be involved in future study to optimize the APPC model.

Recent literature pointed out that HFTS can directly modulate molecules, leading to the regulation of related biological functions [32]. Previous studies indicated that different frequencies of HFTS resonates with various ion channels, such as voltage-gated calcium channels (Ca_v_) and sodium channels (Na_v_), affecting the corresponding ion currents and thereby influencing neuronal excitability [33]. Our previous study showed that HFTS inhibits the enhanced PYR^ACC^ excitability in mice with neuropathic pain by enhancing the Kv conductance [10,34]. The ACC, located beneath the skull, is critically important in both pain-related and stress-induced anxiety [15,35]. It is notable that HFTS exerts an inhibitory effect on both PYR^ACC^ and INT^ACC^ neurons according to in vivo recording. This raises a question: What is the contribution of each type of neuron to the overall inhibitory effect on PYR^ACC^? By performing whole-cell patch-clamp recording, we showed that the E/I ratios were decreased, indicating that the net effect of HFTS on PYR^ACC^ neurons was to reduce their excitability. This investigation also revealed that the anxiolytic effect of HFTS was closely linked with the reduced FOS expression and decreased in vivo firing of PYR in the ACC, which implies that the anxiolytic effect of HFTS arises from inhibiting the excitability of PYR neurons. Further whole-cell patch-clamping experiments further confirmed that the inhibition of PYR neurons is mediated through enhancing the voltage-gated K^+^ and the K2P channels. These results are consistent with our previous computational simulation findings, which reveal that a frequency of ~36 THz corresponds to the carbonyl group in the filter region of Kv1.2 channels and facilitates the conductance of potassium ions [10]. What is more, the amplitudes of both mEPSCs and mIPSCs in PYR^ACC^ neurons were reduced after HFTS (Fig. S3H to M), which indicates that HFTS should directly or indirectly modulate the function of AMPARs and GABA_A_Rs on PYR^ACC^ neurons. Further, we found that HFTS no longer affected the frequency and amplitude of mEPSCs and mIPSCs and the amplitude ratio and frequency ratio of mEPSC/mIPSC in the presence of Kv and K2P channel blockers. This strongly suggests that the effects of HFTS on mEPSCs and mIPSCs are secondary to its enhancement of K^+^ channel activity. We propose that HFTS increases the currents of the K^+^ channel (particularly the Kv channel), which subsequently decreases the amplitudes of mEPSCs and mIPSCs. This proposal is supported by several reported works: (a) Inhibition of Kv4.2-mediated currents increases the amplitude of mEPSCs and excitatory postsynaptic potentials in the hippocampus, by facilitating the recruitment of AMPARs [36,37]. (b) Activation of Kv channels near the postsynaptic areas suppresses the GABAergic inhibition of pyramidal cell dendrites in the hippocampus [38]. (c) Blocking Kv channels enhances the amplitude of inhibitory postsynaptic currents in cerebellar Purkinje and basket cells [39]. Although our experiments confirmed that HFTS directly enhances K^+^ currents and indirectly inhibits the amplitude of mEPSCs/mIPSCs, the mechanisms by which K^+^ channels affect α-amino-3-hydroxy-5-methyl-4-isoxazolepropionic acid and GABA receptors have not been thoroughly investigated, which could be further investigated in future studies.

A limitation of this study is the inability to simultaneously monitor channel-specific currents and behavior due to the technical challenges of in vivo patch-clamp recordings in behaving animals. Moreover, our study focused on the net effects of HFTS on pyramidal neuron activity without examining potential contributions from glial cells. As HFTS is not cell type specific, the observed neuronal changes may integrate both direct neuronal and indirect glial-mediated effects—an important mechanistic question for future cell-specific investigations.

To sum up, this research marks a major advancement in developing innovative physical treatment approaches for stress-related anxiety and central nervous system disorders. The APPC, serving as a classifier to identify anxiety-susceptible mice, holds potential for future studies to assess the efficacy of various treatments.

## Methods

### Animals

C57BL/6J mice (male, 6 to 8 weeks old), weighing between 22 and 26 g, were sourced from the Experimental Animal Center at the Fourth Military Medical University. The mice were housed in a laboratory with controlled environmental conditions: a temperature of 22 to 26 °C, 40% humidity, and 12-h light–dark cycle with illumination from 9:00 AM to 9:00 PM. They had free access to food and water. After a week-long acclimatization period, the mice were randomly divided into 2 groups. All procedures and animal care adhered to the guidelines established by the Ethics Committee of the Fourth Military Medical University.

### Acute restraint stress

To induce ARS, mice were immobilized in 50-ml centrifuge tubes for 2 h. This method restricted major head and limb movements while ensuring ventilation [14]. The procedure was conducted between 8:00 and 10:00 AM. Control mice moved freely in their cages during this period, without access to water or food. After restraint, mice were allowed a brief recovery in their home cages to minimize nonspecific motor effects. Behavioral assays were conducted post-stress to evaluate the effects of ARS in mice.

### Open-field test

The open-field experiment was performed using an apparatus (BioMed Easy Technologies Co., Ltd, Guangdong, China) to evaluate anxiety-related behavior in mice. Individual mice were immediately positioned in the center of the testing arena for unrestricted exploration. Their movements were tracked and recorded using an automated behavioral monitoring system over a standardized 15-min observation period. Between trials, the apparatus was thoroughly sanitized with 75% ethanol to eliminate odor interference, ensuring no cross-test contamination. The software then analyzed the time in the central area and the percentage of central distance by each mouse.

### Elevated plus maze

The study utilized a maze apparatus (BioMed Easy Technologies Co., Ltd, Guangdong, China) composed of a central platform (5 × 5 cm), 2 enclosed arms (30 × 5 × 20 cm), and 2 opposing open arms (30 × 5 cm). Elevated 100 cm above the floor, mice were positioned at the maze center facing the open arms and allowed unrestricted exploration for a predefined period. Posttest, movement trajectories and heat maps were analyzed offline, with quantification of the time spent in the open arms and number of entries into these zones. To mitigate odor-related confounders, the maze was rigorously sanitized with 75% ethanol between trials.

### Light/dark box

The LDB experiment was employed to assess anxiety-like behavior in mice. The apparatus consisted of 2 compartments: a brightly illuminated chamber and a dimly lit chamber, separated by a small opening. Mice were placed in the illuminated chamber to enable unconstrained exploration of both compartments. Behavioral metrics, including locomotion trajectories and time allocation within each compartment, were tracked using an automated system (TrackingMaster, VanBi Technology Inc., Shanghai, China) for post hoc analysis. Key parameters such as total duration in the light chamber and frequency of transitions into this zone were quantified. To prevent inter-trial contamination, the apparatus was rigorously sanitized with 75% ethanol between sessions.

### HFTS and BLS

HFTS was delivered using a quantum cascade laser (center frequency: 35.93 ± 0.1 THz). The laser output was routed through a custom coupler (Innovation Laboratory of Terahertz Biophysics) and integrated with a polycrystalline infrared fiber (Art Photonics) featuring an AgCl/Br core. This fiber demonstrated high transmittance (3- to 18-μm wavelength range), a core refractive index of 2.15, and an effective numerical aperture of 0.35 ± 0.05. For targeted stimulation, the fiber tip (1-cm length, 600-μm diameter) was positioned over the thinned skull of ARS-induced anxiety-susceptible mice, aligned with the following ACC coordinates: 1.10 mm anterior to the bregma and 0.35 mm lateral to the midline. HFTS parameters included a 10-min exposure duration, a 2-μs pulse width, a 10-kHz repetition frequency, and 40% duty cycle. The average power output at the fiber tip, verified using a mid-infrared detector (NOVA II-3A, Israel), was 0.3 ± 0.05 mW. For comparative analysis, the same ACC region was stimulated for 10 min with a blue laser (473-nm wavelength, 5-ms pulse duration, 1-Hz frequency, 10-mW average power).

### Feature selection

By preprocessing and feature extraction of mouse behavioral signals, we obtained sample data containing 6 features. Due to the inherent characteristics of the feature indicators, there are significant differences in their numerical ranges. To reduce the interference of numerical values on model training, each feature is standardized using Eq. (1):

$$X_{\mathrm{ij}}=\frac{x_{\mathrm{ij}}-u_{i}}{\sigma_{i}}$$ (1)

where $X_{\mathrm{ij}}$ is the standardized value, $x_{\mathrm{ij}}$ is the original value of *j* sample under a certain feature, and $u_{i}$ and $\sigma_{i}$ are the mean and standard deviation of each respective feature, respectively. *i* ∈ [1, 6], and *i* is an integer.

Due to the complexity of the experimental procedures for feature extraction and the need to ensure that these features positively impact classification accuracy, feature selection is crucial for enhancing experimental efficiency. In this experiment, a feature selector was employed to identify the most distinctive features. We use the ReliefF algorithm, which effectively evaluates feature importance by calculating weights based on the correlation between each feature and the categories, handling multifeature and multicategory data. It searches for the nearest neighbor of each feature using the Manhattan distance to determine the most significant features for classification [40,41].

### Anxiety phenotype classification

To identify the most effective classifier, a confusion matrix was used to assess the performance of RF and NB. The signals filtered by the optimal feature selector were then input into the best-performing classifier to determine the anxiety status of the mice. RF, an ensemble model based on decision trees, aggregates outcomes through majority voting to mitigate overfitting and handle high-dimensional, nonlinear data effectively [42]. NB, a probabilistic classification method rooted in Bayes’s theorem, assumes feature independence and performs well even with limited data, exhibiting excellent performance in large-scale classification tasks [43].

### Transmittance test of mouse skulls

Spectral analysis of thinned mouse skulls was conducted using a Nicolet 6700 Fourier transform infrared microspectrometer (Thermo Scientific) in transmission mode on the BL01B1 beamline at the National Center for Protein Science Shanghai [44]. Measurements were acquired through 64 co-added scans at a 4 cm^−1^ spectral resolution, covering the mid-infrared region from 800 to 4,000 cm^−1^, using an 80-mm diaphragm aperture setting. The skull region of interest, isolated to a diameter of approximately 1 cm, targeted the ACC at stereotaxic coordinates of 1.10 mm anterior to the bregma and 0.35 mm lateral to the midline on the right hemisphere.

### Immunohistochemical staining

Immunohistochemical staining for FOS in the ACC region was performed to assess changes in the number of activated neurons as described in our previous studies [45]. Mice were subjected to either HFTS (36 THz, 10 min) or BLS (1 Hz, 10 min) and then deeply anesthetized and perfused initially with saline, followed by paraformaldehyde. The brains were then removed, placed in a sucrose-containing solution, and sectioned using a freezing microtome into serial frontal slices. The sections were incubated for 48 h with a primary antibody against FOS (1:400, ab208942, Abcam, MA, USA), diluted in a buffer containing normal donkey serum and Triton X-100. After washing, the sections were treated with appropriate fluorophore-conjugated secondary antibodies (1:400) for 4 h at room temperature. 4′,6-Diamidino-2-phenylindole was used to counterstain cell nuclei, aiding in the identification of FOS^+^ cells. Imaging was conducted using a confocal microscope (FV1000, Olympus, Japan) or a slide scanner (Slideview, VS200, Olympus, Japan). Counting was performed by an investigator who was blinded to the treatment conditions.

Then, immunofluorescence staining for FOS/CaMKII/GABA in the ACC region was performed to evaluate the specific regulatory effect of terahertz waves on neurons. The brain slices from the sham, the ARS, the ARS + BLS, and the ARS + HFTS group were incubated for 48 h with mouse anti-FOS (1:400, ab208942, Abcam, MA, USA), rabbit anti-CaMKII (1:500, ab216503, Abcam, MA, USA), and guinea pig anti-GABA antibodies (1:200, ab17413, Abcam, MA, USA). After washing, the sections were treated with appropriate fluorophore-conjugated secondary antibodies (1:400, Invitrogen, Thermo Fisher, CA, USA) for 4 h at room temperature. Imaging was conducted by using a confocal microscope (FV1000, Olympus, Japan). Counting was performed by an investigator who was blinded to the treatment conditions.

### In vitro patch-clamp recording

Mice were anesthetized and euthanized via decapitation. Coronal brain slices (300-μm thickness) containing the ACC were prepared using a vibrating microtome (Leica VT 1200s, Germany) in ice-cold (0 to 4 °C), oxygenated artificial cerebrospinal fluid (ACSF; 95% O_2_/5% CO_2_). The ACSF composition (mM) was as follows: 124 NaCl, 25 NaHCO_3_, 2.5 KCl, 1 NaH_2_PO_4_, 2 CaCl_2_, 1 MgSO_4_, and 10 glucose [10,45]. Neurons were visualized using infrared differential interference contrast or fluorescence microscopy (Olympus BX51W1, Japan). Patch pipettes (2- to 5-MΩ resistance) were filled with an intracellular solution containing (mM) the following: 124 K-gluconate, 5 NaCl, 1 MgCl_2_, 0.2 EGTA, 2 MgATP, 0.1 Na_3_GTP, 10 HEPES, and 10 phosphocreatine (pH 7.3 adjusted with KOH; 290 mOsm). Once the neurons had been patched, we applied a current stimulus (400 ms, 200 pA) to the neuron in current-clamp mode. Those neurons with typical irregular firing patterns, a larger membrane capacitance (Cs > 100 pf), and a smaller membrane resistance (Ra < 200 MΩ) were classified as PYR^ACC^ neurons and qualified for subsequent electrophysiological experiments (Fig. S3B) [46,47]. To isolate voltage-gated Kv currents, 1 μM TTX and 100 μM CdCl_2_ were added to ACSF to block Na^+^ and Ca^2+^ channels, respectively. For K2P current recordings, 4 mM BaCl_2_ was applied to inhibit most K2P channel subtypes. TTX (1 μM) was added to the ACSF for recording mEPSCs and mIPSCs. To study the in vitro effects of HFTS and BLS, we applied them separately to the ACC region on the brain slices of ARS or sham-treated mice. For mEPSC and mIPSC recordings, the cells were voltage-clamped at −70 or 0 mV, and to block Kv channels and K2P channels, 4-AP (3 mM) and BaCl_2_ (4 mM) were added into the ACSF. The data were analyzed with the Mini Analysis software (Synaptosoft Inc., Fort Lee, NJ, USA). To record eEPSCs and eIPSCs, a bipolar platinum electrode was placed at the ACC. The eEPSC paired-pulse ratio (eEPSC-PPR) and eIPSC-PPR were detected by paired electrical stimulation. The eEPSC-PPR and eIPSC-PPR were calculated as the ratio of the peak current response to the second pulse divided by that to the first pulse. For checking the E/I ratio, mEPSC/mIPSC and eEPSC/eIPSC were recorded from the same neuron. In this case, the patch pipettes were filled with an intracellular solution containing (mM) the following: CsMeSO_3_ (122), NaCl (3.7), HEPES (20), BAPTA (10), EGTA (0.2), MgATP (0.3), Na_3_GTP (0.3), TEA-Cl (5), Qx314-Br (5), and spermine (0.1). Signals were amplified (MultiClamp 700B, Molecular Devices, USA), low-pass filtered at 1 kHz, and digitized at 10 kHz (Axon DigiData 1550A). Data were analyzed offline using the Clampfit 10.02 software.

### In vivo multichannel recording

Prior to the recording procedure, we implanted custom-designed electrodes into the right ACC of the mice, adhering to the stereotaxic coordinates detailed in our earlier reports [48]: 1.1 mm anterior to the bregma, 0.3 mm lateral to the midline (right), and 1.8 mm vertical to the skull surface. The electrode assembly, consisting of a 16-channel wire array with a central hollow tube, was affixed to the exposed skull using dental adhesive resin cement (Super-Bond C&B, Japan) [10]. The single-unit recording was performed by multichannel recording technology provided by the Neurolego system (Nanjing Greathink Medical Technology, Nanjing, China). Following this, single-unit spike sorting was conducted using the MClust-v4.4 toolbox in the MATLAB software (MathWorks, USA). Within the ACC, 2 principal neuronal subtypes were identified: pyramidal neurons exhibiting long-duration APs (>430 μs) and GABAergic interneurons characterized by shorter durations (<430 μs) [49].

### Statistical analysis

The present study was conducted with strict blinding for all experiments and data analyses. GraphPad Prism (v 9.5.1) was used for statistical evaluations and graph creation. Statistical significance was determined using paired or unpaired *t* tests, 1-way analysis of variance (ANOVA), 2-way repeated-measures ANOVA, followed by the Holm–Šídák test for post hoc comparisons, and Wilcoxon matched-paired signed rank test. Results are presented as mean ± standard error of the mean, with statistical significance denoted as follows: *P < 0.05, **P < 0.01, ***P < 0.001, ****P < 0.0001, and ns (not significant) for *P* > 0.05.

## Data Availability

The datasets used and/or analyzed during the present study are available from the corresponding authors on reasonable request.
