# Supplementary material for: Transcranial High-Frequency Terahertz Stimulation Alleviates Anxiety-like Behavior in Mice via a Noninvasive Approach
Source: Research (Wash D C). 2025 Aug 8;8:0766. doi: 10.34133/research.0766 (PMC12332262; doi:10.34133/research.0766)
Supplement: Supplementary 1 — Figs. S1 to S3 Tables S1 to S10 [file research.0766.f1.zip › Supplemental Material.docx]

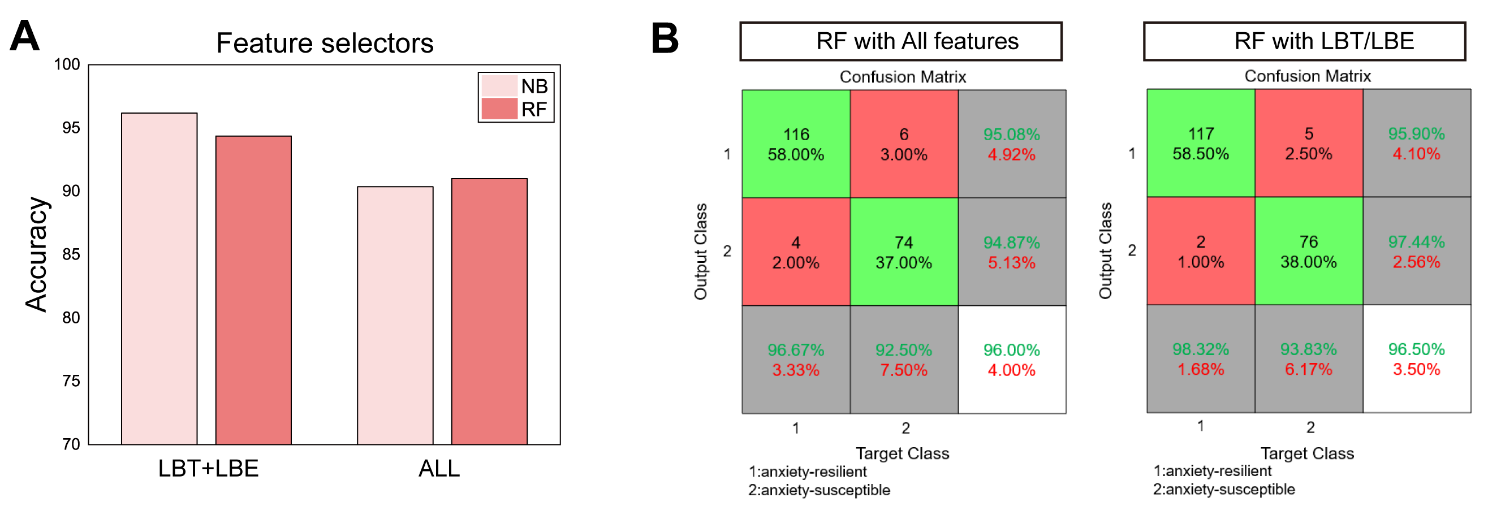


**Fig. S1. Feature selection and model selection for Anxiety Phenotype Prediction Classifier.** (A) Comparison of the accuracy of Naïve Bayes and Random Forest with two features/all features. (B) Left: Confusion matrixes of Random Forest (RF) with all features. Right: Confusion matrixes of Random Forest (RF) with two features.


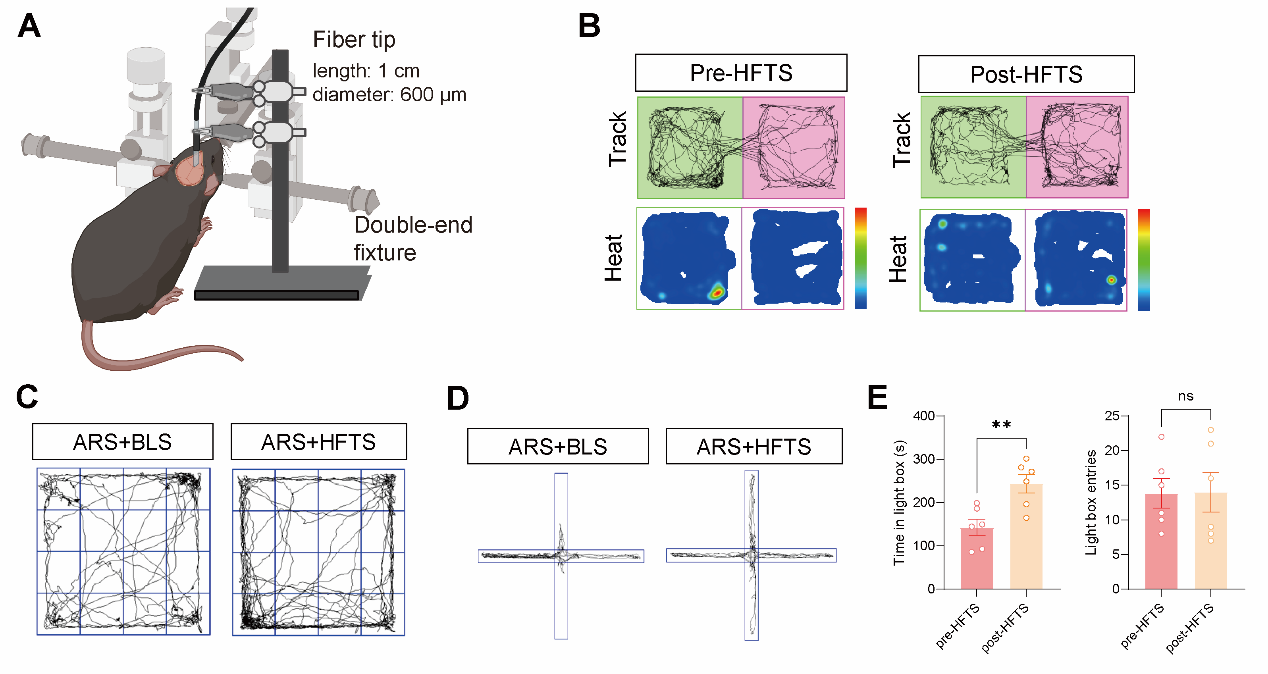


**Fig. S2 The anxiolytic effect of non-invasive HFTS in light/dark test, open field test, and elevated plus maze test.** (A) The schematic illustration of fiber fixation by a double-end fixture of transcranial HFTS application. (B) Representative track and heat map of light/dark test pre-/post-HFTS. (C) Representative track of open field test in ARS+BLS and ARS+HFTS group. (D) Representative track of elevated plus maze test in ARS+BLS and ARS+HFTS group. (E) Histograms showing the behavioral changes in LDB test after non-invasive HFTS application of anxiety-susceptible mice. Left: summarized data showing light box time (LBT) before and after HFTS in ARS-susceptible mice (pre-HFTS vs. post-HFTS: *p* = 0.0081). Right: summarized data showing Light Box Entries (LBE) after HFTS/BLS in ARS mice (pre-HFTS vs. post-HFTS: *p* = 0.9099), n_pre-HFTS_ = 6, n_post-HFTS_ = 6, paired *t*-test. ***p* < 0.01, ns, *p* > 0.05.


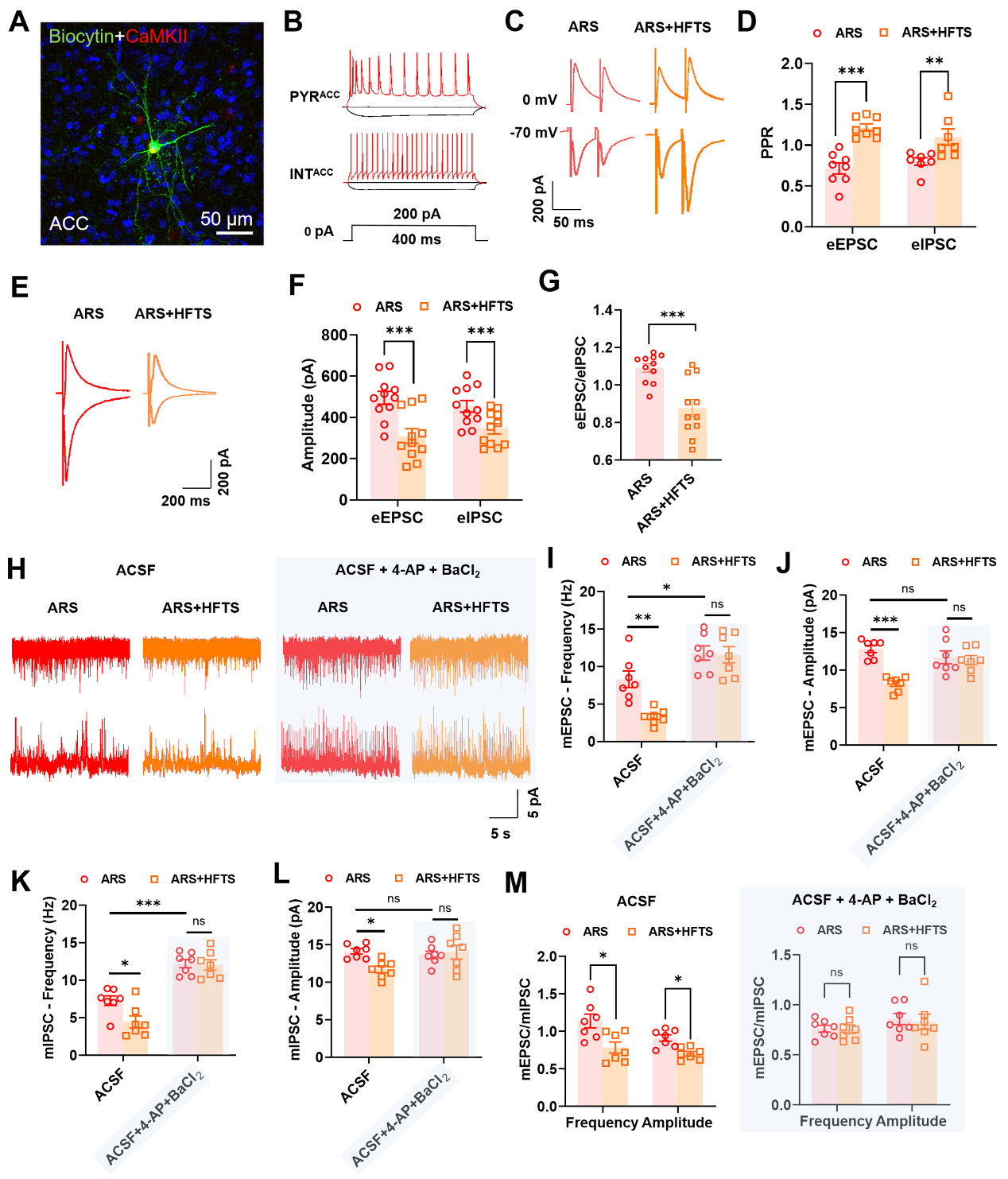


**Fig. S3 HFTS decreases the synaptic inputs of PYR^ACC^ neurons of ARS mice.**

(A) Representative images showing the recorded neurons labeled with cy3 (red) and injected with biocytin (green) in the ACC. Scale bar, 50 μm. (B) Representative firing patterns of PYR^ACC^ neurons and INT^ACC^ neurons. (C-D) Representative traces (C) and summarized results (D) of the eEPSC PPR and eIPSC PPR before and after HFTS in ARS mice (n_eEPSC_ = 8 neurons, n_eIPSC_ = 7 neurons from 3 mice). (E-F) Representative traces (E) and summarized results (F) of the amplitude of paired eEPSC and eIPSC before and after HFTS in ARS mice (n = 11 neurons from 3 mice). (G) The eEPSC/eIPSC ratio before and after HFTS in ARS mice. (H) Representative traces of paired mEPSCs and mIPSCs of PYR^ACC^ neurons before and after HFTS in ARS mice with or without 4-AP and BaCl_2_ (n = 7 neurons from 3 mice). (I-J) Summarized results of the frequency (I) and amplitude (J) of mEPSCs. (K-L) Summarized results of the frequency (K) and amplitude (L) of mIPSCs. (M) Ratio of mEPSC/mIPSC before and after HFTS in ARS mice, with or without 4-AP and BaCl_2_. **p* < 0.05, ****p* < 0.001, ns *p* > 0.05.
