# Supplementary material for: Transcranial High-Frequency Terahertz Stimulation Alleviates Anxiety-like Behavior in Mice via a Noninvasive Approach
Source: Research (Wash D C). 2025 Aug 8;8:0766. doi: 10.34133/research.0766 (PMC12332262; doi:10.34133/research.0766)
Supplement: Supplementary 1 — Figs. S1 to S3 Tables S1 to S10 [file research.0766.f1.zip › Table S1-S10.docx]

| **Table S1.** The spike frequency in PNs of ACC (sham vs. ARS mice) | | | | | |
| --- | --- | --- | --- | --- | --- |
|  |  |  |  | Related to Figure2 |  |
| Table Analyzed | Sham vs ARS |  |  |  |  |
|  |  |  |  |  |  |
| Two-way ANOVA | Ordinary |  |  |  |  |
| Alpha | 0.05 |  |  |  |  |
|  |  |  |  |  |  |
| Source of Variation | % of total variation | P value | P value summary | Significant? |  |
| Interaction | 1.766 | 0.0412 | * | Yes |  |
| Row Factor | 77.33 | <0.0001 | **** | Yes |  |
| Column Factor | 8.862 | <0.0001 | **** | Yes |  |
|  |  |  |  |  |  |
| ANOVA table | SS | DF | MS | F (DFn, DFd) | P value |
| Interaction | 320.8 | 11 | 29.17 | F (11, 144) = 1.920 | P=0.0412 |
| Row Factor | 14045 | 11 | 1277 | F (11, 144) = 84.05 | P<0.0001 |
| Column Factor | 1610 | 1 | 1610 | F (1, 144) = 106.0 | P<0.0001 |
| Residual | 2188 | 144 | 15.19 |  |  |
|  |  |  |  |  |  |
| Difference between column means |  |  |  |  |  |
| Mean of Sham | 10.92 |  |  |  |  |
| Mean of ARS | 17.11 |  |  |  |  |
| Difference between means | -6.19 |  |  |  |  |
| SE of difference | 0.6014 |  |  |  |  |
| 95% CI of difference | -7.379 to -5.002 |  |  |  |  |
|  |  |  |  |  |  |
| Data summary |  |  |  |  |  |
| Number of columns (Column Factor) | 2 |  |  |  |  |
| Number of rows (Row Factor) | 12 |  |  |  |  |
| Number of values | 168 |  |  |  |  |

| **Table S2**. The Kv current to different voltage in PNs of ACC (sham vs. ARS mice) | | | | | |
| --- | --- | --- | --- | --- | --- |
|  |  |  |  | Related to Figure2 |  |
| sham - ARS | Mean Diff. | 95.00% CI of diff. | Below threshold? | Summary | Adjusted P Value |
| -70 mV | 1.538 | -43.04 to 46.11 | No | ns | >0.9999 |
| -60 mV | 1.689 | -42.88 to 46.26 | No | ns | >0.9999 |
| -50 mV | 1.599 | -42.97 to 46.17 | No | ns | >0.9999 |
| -40 mV | 1.906 | -42.67 to 46.48 | No | ns | >0.9999 |
| -30 mV | 2.341 | -42.23 to 46.91 | No | ns | >0.9999 |
| -20 mV | 3.487 | -41.09 to 48.06 | No | ns | >0.9999 |
| -10 mV | 5.863 | -38.71 to 50.44 | No | ns | >0.9999 |
| 0 mV | 9.889 | -34.68 to 54.46 | No | ns | >0.9999 |
| 10 mV | 14.35 | -30.23 to 58.92 | No | ns | >0.9999 |
| 20 mV | 19.6 | -24.98 to 64.17 | No | ns | 0.9943 |
| 30 mV | 24.89 | -19.68 to 69.46 | No | ns | 0.9051 |
| 40 mV | 30.49 | -14.08 to 75.07 | No | ns | 0.6035 |
| 50 mV | 36.42 | -8.157 to 80.99 | No | ns | 0.2611 |
| 60 mV | 40.74 | -3.835 to 85.31 | No | ns | 0.1152 |
| 70 mV | 45.2 | 0.6270 to 89.77 | Yes | * | 0.0433 |
| 80 mV | 50.4 | 5.823 to 94.97 | Yes | * | 0.0121 |
| 90 mV | 54.9 | 10.33 to 99.48 | Yes | ** | 0.0036 |
| 100 mV | 60.21 | 15.64 to 104.8 | Yes | *** | 0.0008 |
| 110 mV | 64.73 | 20.15 to 109.3 | Yes | *** | 0.0002 |
| 120 mV | 69.29 | 24.71 to 113.9 | Yes | **** | <0.0001 |
| 130 mV | 71.88 | 27.31 to 116.5 | Yes | **** | <0.0001 |
| 140 mV | 76.98 | 32.41 to 121.6 | Yes | **** | <0.0001 |
| 150 mV | 79.79 | 35.21 to 124.4 | Yes | **** | <0.0001 |
| 160 mV | 83.24 | 38.66 to 127.8 | Yes | **** | <0.0001 |
| 170 mV | 90.58 | 46.01 to 135.2 | Yes | **** | <0.0001 |
| 180 mV | 95.72 | 51.14 to 140.3 | Yes | **** | <0.0001 |
| 190 mV | 100.7 | 56.11 to 145.3 | Yes | **** | <0.0001 |
| 200 mV | 106.3 | 61.74 to 150.9 | Yes | **** | <0.0001 |
|  |  |  |  |  |  |
| Source of Variation | % of total variation | P value | P value summary | Significant? |  |
| Interaction | 4.772 | <0.0001 | **** | Yes |  |
| Row Factor | 77.64 | <0.0001 | **** | Yes |  |
| Column Factor | 7.915 | <0.0001 | **** | Yes |  |
|  |  |  |  |  |  |
| ANOVA table | SS (Type III) | DF | MS | F (DFn, DFd) | P value |
| Interaction | 148268 | 27 | 5491 | F (27, 448) = 6.113 | P<0.0001 |
| Row Factor | 2412221 | 27 | 89342 | F (27, 448) = 99.45 | P<0.0001 |
| Column Factor | 245914 | 1 | 245914 | F (1, 448) = 273.7 | P<0.0001 |
| Residual | 402450 | 448 | 898.3 |  |  |
|  |  |  |  |  |  |
| Difference between column means |  |  |  |  |  |
| Predicted (LS) mean of Sham | 109.8 |  |  |  |  |
| Predicted (LS) mean of ARS | 65.35 |  |  |  |  |
| Difference between predicted means | 44.45 |  |  |  |  |
| SE of difference | 2.687 |  |  |  |  |
| 95% CI of difference | 39.17 to 49.73 |  |  |  |  |
|  |  |  |  |  |  |
| Data summary |  |  |  |  |  |
| Number of columns (Column Factor) | 2 |  |  |  |  |
| Number of rows (Row Factor) | 28 |  |  |  |  |
| Number of values | 504 |  |  |  |  |

| **Table S3**. The K2P current to different voltage in PNs of ACC (sham vs. ARS mice) | | | | | |
| --- | --- | --- | --- | --- | --- |
|  |  |  |  | Related to Figure2 |  |
| sham - ARS | Mean Diff. | 95.00% CI of diff. | Below threshold? | Summary | Adjusted P Value |
| -120 mV | -43.67 | -112.0 to 24.70 | No | ns | 0.5253 |
| -110 mV | -26.78 | -95.16 to 41.59 | No | ns | 0.9559 |
| -100 mV | -13.17 | -81.55 to 55.20 | No | ns | 0.9999 |
| -90 mV | -0.4055 | -68.78 to 67.97 | No | ns | >0.9999 |
| -80 mV | 7.236 | -61.14 to 75.61 | No | ns | >0.9999 |
| -70 mV | 13.28 | -55.10 to 81.65 | No | ns | 0.9998 |
| -60 mV | 16.34 | -52.04 to 84.72 | No | ns | 0.999 |
| -50 mV | 25.04 | -43.34 to 93.41 | No | ns | 0.9719 |
| -40 mV | 46.41 | -21.97 to 114.8 | No | ns | 0.4373 |
| -30 mV | 99.84 | 31.47 to 168.2 | Yes | *** | 0.0005 |
|  |  |  |  |  |  |
| Source of Variation | % of total variation | P value | P value summary | Significant? |  |
| Interaction | 3.554 | 0.0047 | ** | Yes |  |
| Row Factor | 73.16 | <0.0001 | **** | Yes |  |
| Column Factor | 0.3775 | 0.1053 | ns | No |  |
|  |  |  |  |  |  |
| ANOVA table | SS (Type III) | DF | MS | F (DFn, DFd) | P value |
| Interaction | 68697 | 9 | 7633 | F (9, 170) = 2.773 | P=0.0047 |
| Row Factor | 1414104 | 9 | 157123 | F (9, 170) = 57.08 | P<0.0001 |
| Column Factor | 7297 | 1 | 7297 | F (1, 170) = 2.651 | P=0.1053 |
| Residual | 467940 | 170 | 2753 |  |  |
|  |  |  |  |  |  |
| Difference between column means |  |  |  |  |  |
| Predicted (LS) mean of Sham | -21.75 |  |  |  |  |
| Predicted (LS) mean of ARS | -34.16 |  |  |  |  |
| Difference between predicted means | 12.41 |  |  |  |  |
| SE of difference | 7.623 |  |  |  |  |
| 95% CI of difference | -2.637 to 27.46 |  |  |  |  |
|  |  |  |  |  |  |
| Data summary |  |  |  |  |  |
| Number of columns (Column Factor) | 2 |  |  |  |  |
| Number of rows (Row Factor) | 10 |  |  |  |  |
| Number of values | 190 |  |  |  |  |

| **Table S4.** HFTS decreases the synaptic inputs of PYR^ACC^ neurons of ARS mice.  Related to Figure S3 | | | | | |
| --- | --- | --- | --- | --- | --- |
|  | Y-axis | eEPSC | | eIPSC | |
|  |  | ARS | ARS+HFTS | ARS | ARS+HFTS |
| Fig. S3 D | PPR | 0.72 ± 0.06 | 1.22 ± 0.04 | 0.80 ± 0.04 | 1.10 ± 0.09 |
| Fig. S3 F | Amplitude (pA) | 494.93 ± 29.74 | 309.62 ± 33.87 | 453.94 ± 26.96 | 344.19 ± 23.40 |
|  | | | | | |
|  |  | ARS | | ARS+HFTS | |
| Fig. S3 G | eEPSC/eIPSC | 1.09 ± 0.02 | | 0.88 ± 0.04 | |
|  | | | | | |
|  |  | ACSF | | ACSF + 4-AP + BaCl_2_ | |
|  |  | ARS | ARS+HFTS | ARS | ARS+HFTS |
| Fig. S3 I | mEPSC-Frequency (Hz) | 8.31 ± 1.02 | 3.28 ± 0.35 | 11.81 ± 0.88 | 11.57 ± 1.00 |
| Fig. S3 J | mEPSC-Amplitude (pA) | 12.83 ± 0.43 | 8.21 ± 0.34 | 11.72 ± 0.80 | 11.34 ± 0.57 |
| Fig. S3 K | mIPSC-Frequency (Hz) | 7.30 ± 0.59 | 4.46 ± 0.74 | 12.23 ± 0.51 | 12.06 ± 0.63 |
| Fig. S3 L | mIPSC-Amplitude (pA) | 14.11 ± 0.33 | 11.71 ± 0.41 | 13.64 ± 0.46 | 14.01 ± 0.85 |
|  | | | | | |
|  |  | ACSF | | ACSF + 4-AP + BaCl_2_ | |
|  |  | ARS | ARS+HFTS | ARS | ARS+HFTS |
| Fig. S3 M | mE/I raio of Frequency | 0.75 ± 0.06 | 1.00 ± 0.17 | 0.79 ± 0.07 | 0.76 ± 0.04 |
|  | mE/I raio of Amplitude | 0.92 ± 0.02 | 0.76 ± 0.04 | 0.70 ± 0.03 | 0.83 ± 0.07 |

| **Table S5**. The spike frequency in PNs of ACC before and after BLS | | | | | |
| --- | --- | --- | --- | --- | --- |
|  |  |  |  | Related to Figure7 |  |
| Table Analyzed | pre BLS vs post BLS |  |  |  |  |
|  |  |  |  |  |  |
| Two-way ANOVA | Ordinary |  |  |  |  |
| Alpha | 0.05 |  |  |  |  |
|  |  |  |  |  |  |
| Source of Variation | % of total variation | P value | P value summary | Significant? |  |
| Interaction | 0.4126 | 0.9848 | ns | No |  |
| Row Factor | 80.42 | <0.0001 | **** | Yes |  |
| Column Factor | 1.209 | 0.0022 | ** | Yes |  |
|  |  |  |  |  |  |
| ANOVA table | SS | DF | MS | F (DFn, DFd) | P value |
| Interaction | 107.4 | 11 | 9.767 | F (11, 144) = 0.3007 | P=0.9848 |
| Row Factor | 20939 | 11 | 1904 | F (11, 144) = 58.61 | P<0.0001 |
| Column Factor | 314.9 | 1 | 314.9 | F (1, 144) = 9.695 | P=0.0022 |
| Residual | 4677 | 144 | 32.48 |  |  |
|  |  |  |  |  |  |
| Difference between column means |  |  |  |  |  |
| Mean of pre-BLS | 17.35 |  |  |  |  |
| Mean of post-BLS | 14.61 |  |  |  |  |
| Difference between means | 2.738 |  |  |  |  |
| SE of difference | 0.8794 |  |  |  |  |
| 95% CI of difference | 1.000 to 4.476 |  |  |  |  |
|  |  |  |  |  |  |
| Data summary |  |  |  |  |  |
| Number of columns (Column Factor) | 2 |  |  |  |  |
| Number of rows (Row Factor) | 12 |  |  |  |  |
| Number of values | 168 |  |  |  |  |

| **Table S6**. The spike frequency in PNs of ACC before and after HFTS | | | | | |
| --- | --- | --- | --- | --- | --- |
|  |  |  |  | Related to Figure7 |  |
| Table Analyzed | pre HFTS vs post HFTS |  |  |  |  |
|  |  |  |  |  |  |
| Two-way ANOVA | Ordinary |  |  |  |  |
| Alpha | 0.05 |  |  |  |  |
|  |  |  |  |  |  |
| Source of Variation | % of total variation | P value | P value summary | Significant? |  |
| Interaction | 4.111 | 0.0427 | * | Yes |  |
| Row Factor | 54.11 | <0.0001 | **** | Yes |  |
| Column Factor | 13.58 | <0.0001 | **** | Yes |  |
|  |  |  |  |  |  |
| ANOVA table | SS | DF | MS | F (DFn, DFd) | P value |
| Interaction | 554.9 | 11 | 50.45 | F (11, 144) = 1.908 | P=0.0427 |
| Row Factor | 7304 | 11 | 664 | F (11, 144) = 25.11 | P<0.0001 |
| Column Factor | 1833 | 1 | 1833 | F (1, 144) = 69.35 | P<0.0001 |
| Residual | 3807 | 144 | 26.44 |  |  |
|  |  |  |  |  |  |
| Difference between column means |  |  |  |  |  |
| Mean of pre-HFTS | 11.99 |  |  |  |  |
| Mean of post-HFTS | 5.387 |  |  |  |  |
| Difference between means | 6.607 |  |  |  |  |
| SE of difference | 0.7934 |  |  |  |  |
| 95% CI of difference | 5.039 to 8.175 |  |  |  |  |
|  |  |  |  |  |  |
| Data summary |  |  |  |  |  |
| Number of columns (Column Factor) | 2 |  |  |  |  |
| Number of rows (Row Factor) | 12 |  |  |  |  |
| Number of values | 168 |  |  |  |  |

| **Table S7**. The Kv current to different voltage in PNs of ARS mice before and after BLS | | | | | |
| --- | --- | --- | --- | --- | --- |
|  |  |  |  | Related to Figure7 |  |
| ARS - ARS+BLS | Mean Diff. | 95.00% CI of diff. | Below threshold? | Summary | Adjusted P Value |
| -70 mV | -0.2045 | -34.76 to 34.35 | No | ns | >0.9999 |
| -60 mV | -0.3657 | -34.92 to 34.19 | No | ns | >0.9999 |
| -50 mV | -0.1638 | -34.72 to 34.39 | No | ns | >0.9999 |
| -40 mV | -0.06095 | -34.61 to 34.49 | No | ns | >0.9999 |
| -30 mV | -0.1455 | -34.70 to 34.41 | No | ns | >0.9999 |
| -20 mV | -0.4475 | -35.00 to 34.11 | No | ns | >0.9999 |
| -10 mV | -1.164 | -35.72 to 33.39 | No | ns | >0.9999 |
| 0 mV | -2.725 | -37.28 to 31.83 | No | ns | >0.9999 |
| 10 mV | -4.492 | -39.04 to 30.06 | No | ns | >0.9999 |
| 20 mV | -6.45 | -41.00 to 28.10 | No | ns | >0.9999 |
| 30 mV | -7.036 | -41.59 to 27.52 | No | ns | >0.9999 |
| 40 mV | -8.393 | -42.95 to 26.16 | No | ns | >0.9999 |
| 50 mV | -9.516 | -44.07 to 25.04 | No | ns | >0.9999 |
| 60 mV | -10.94 | -45.49 to 23.61 | No | ns | >0.9999 |
| 70 mV | -10.99 | -45.55 to 23.56 | No | ns | >0.9999 |
| 80 mV | -12.02 | -46.57 to 22.53 | No | ns | 0.9999 |
| 90 mV | -12.89 | -47.44 to 21.67 | No | ns | 0.9996 |
| 100 mV | -13.24 | -47.79 to 21.31 | No | ns | 0.9993 |
| 110 mV | -15.1 | -49.65 to 19.45 | No | ns | 0.9946 |
| 120 mV | -15.48 | -50.03 to 19.07 | No | ns | 0.9924 |
| 130 mV | -16.6 | -51.15 to 17.95 | No | ns | 0.9808 |
| 140 mV | -17.3 | -51.85 to 17.25 | No | ns | 0.9686 |
| 150 mV | -17.74 | -52.29 to 16.82 | No | ns | 0.9585 |
| 160 mV | -18.16 | -52.71 to 16.39 | No | ns | 0.9465 |
| 170 mV | -18.86 | -53.41 to 15.69 | No | ns | 0.9218 |
| 180 mV | -19.32 | -53.88 to 15.23 | No | ns | 0.9019 |
| 190 mV | -19.39 | -53.94 to 15.16 | No | ns | 0.8986 |
| 200 mV | -18.59 | -53.14 to 15.97 | No | ns | 0.9322 |
|  |  |  |  |  |  |
| Source of Variation | % of total variation | P value | P value summary | Significant? |  |
| Interaction | 0.3247 | 0.9951 | ns | No |  |
| Row Factor | 91.14 | <0.0001 | **** | Yes |  |
| Column Factor | 0.644 | <0.0001 | **** | Yes |  |
|  |  |  |  |  |  |
| ANOVA table | SS | DF | MS | F (DFn, DFd) | P value |
| Interaction | 4168 | 27 | 154.4 | F (27, 280) = 0.4268 | P=0.9951 |
| Row Factor | 1169940 | 27 | 43331 | F (27, 280) = 119.8 | P<0.0001 |
| Column Factor | 8267 | 1 | 8267 | F (1, 280) = 22.85 | P<0.0001 |
| Residual | 101290 | 280 | 361.8 |  |  |
|  |  |  |  |  |  |
| Difference between column means |  |  |  |  |  |
| Mean of pre-BLS | 69.49 |  |  |  |  |
| Mean of post-BLS | 79.41 |  |  |  |  |
| Difference between means | -9.921 |  |  |  |  |
| SE of difference | 2.075 |  |  |  |  |
| 95% CI of difference | -14.01 to -5.836 |  |  |  |  |
|  |  |  |  |  |  |
| Data summary |  |  |  |  |  |
| Number of columns (Column Factor) | 2 |  |  |  |  |
| Number of rows (Row Factor) | 28 |  |  |  |  |
| Number of values | 336 |  |  |  |  |

| **Table S8**. The Kv current to different voltage in PNs of ARS mice before and after HFTS | | | | | |
| --- | --- | --- | --- | --- | --- |
|  |  |  |  | Related to Figure7 |  |
| ARS - ARS+HFTS | Mean Diff. | 95.00% CI of diff. | Below threshold? | Summary | Adjusted P Value |
| -70 mV | -0.7144 | -50.83 to 49.40 | No | ns | >0.9999 |
| -60 mV | -0.4786 | -50.60 to 49.64 | No | ns | >0.9999 |
| -50 mV | -0.437 | -50.55 to 49.68 | No | ns | >0.9999 |
| -40 mV | -0.1923 | -50.31 to 49.92 | No | ns | >0.9999 |
| -30 mV | -0.8092 | -50.93 to 49.31 | No | ns | >0.9999 |
| -20 mV | -2.087 | -52.20 to 48.03 | No | ns | >0.9999 |
| -10 mV | -3.591 | -53.71 to 46.53 | No | ns | >0.9999 |
| 0 mV | -5.158 | -55.28 to 44.96 | No | ns | >0.9999 |
| 10 mV | -7.484 | -57.60 to 42.63 | No | ns | >0.9999 |
| 20 mV | -10.29 | -60.41 to 39.83 | No | ns | >0.9999 |
| 30 mV | -13.5 | -63.62 to 36.62 | No | ns | >0.9999 |
| 40 mV | -17.21 | -67.33 to 32.90 | No | ns | >0.9999 |
| 50 mV | -21.75 | -71.87 to 28.36 | No | ns | 0.9952 |
| 60 mV | -27.04 | -77.16 to 23.08 | No | ns | 0.9309 |
| 70 mV | -31.64 | -81.76 to 18.48 | No | ns | 0.7488 |
| 80 mV | -36.62 | -86.74 to 13.50 | No | ns | 0.4685 |
| 90 mV | -41.74 | -91.86 to 8.379 | No | ns | 0.23 |
| 100 mV | -46.99 | -97.11 to 3.123 | No | ns | 0.0921 |
| 110 mV | -52.92 | -103.0 to -2.806 | Yes | * | 0.0278 |
| 120 mV | -58.91 | -109.0 to -8.791 | Yes | ** | 0.0072 |
| 130 mV | -62.52 | -112.6 to -12.40 | Yes | ** | 0.003 |
| 140 mV | -68.34 | -118.5 to -18.22 | Yes | *** | 0.0007 |
| 150 mV | -72.78 | -122.9 to -22.66 | Yes | *** | 0.0002 |
| 160 mV | -76.29 | -126.4 to -26.18 | Yes | **** | <0.0001 |
| 170 mV | -84.92 | -135.0 to -34.81 | Yes | **** | <0.0001 |
| 180 mV | -91.04 | -141.2 to -40.92 | Yes | **** | <0.0001 |
| 190 mV | -99.12 | -149.2 to -49.00 | Yes | **** | <0.0001 |
| 200 mV | -107.2 | -157.4 to -57.13 | Yes | **** | <0.0001 |
|  |  |  |  |  |  |
| Source of Variation | % of total variation | P value | P value summary | Significant? |  |
| Interaction | 3.98 | <0.0001 | **** | Yes |  |
| Row Factor | 80.66 | <0.0001 | **** | Yes |  |
| Column Factor | 4.79 | <0.0001 | **** | Yes |  |
|  |  |  |  |  |  |
| ANOVA table | SS | DF | MS | F (DFn, DFd) | P value |
| Interaction | 112741 | 27 | 4176 | F (27, 336) = 4.688 | P<0.0001 |
| Row Factor | 2284656 | 27 | 84617 | F (27, 336) = 94.99 | P<0.0001 |
| Column Factor | 135676 | 1 | 135676 | F (1, 336) = 152.3 | P<0.0001 |
| Residual | 299298 | 336 | 890.8 |  |  |
|  |  |  |  |  |  |
| Difference between column means |  |  |  |  |  |
| Mean of pre-HFTS | 76.47 |  |  |  |  |
| Mean of post-HFTS | 113.7 |  |  |  |  |
| Difference between means | -37.21 |  |  |  |  |
| SE of difference | 3.015 |  |  |  |  |
| 95% CI of difference | -43.14 to -31.28 |  |  |  |  |
|  |  |  |  |  |  |
| Data summary |  |  |  |  |  |
| Number of columns (Column Factor) | 2 |  |  |  |  |
| Number of rows (Row Factor) | 28 |  |  |  |  |
| Number of values | 392 |  |  |  |  |

| **Table S9**. The K2P current to different voltage in PNs of ARS mice before and after HFTS (ARS vs. ARS+HFTS mice) | | | | | |
| --- | --- | --- | --- | --- | --- |
|  |  |  |  | Related to Figure7 |  |
| ARS - ARS+HFTS | Mean Diff. | 95.00% CI of diff. | Below threshold? | Summary | Adjusted P Value |
| -120 mV | 67.28 | -22.32 to 156.9 | No | ns | 0.2943 |
| -110 mV | 52.34 | -37.26 to 141.9 | No | ns | 0.6447 |
| -100 mV | 39.25 | -50.35 to 128.9 | No | ns | 0.9099 |
| -90 mV | 27.53 | -62.07 to 117.1 | No | ns | 0.992 |
| -80 mV | 14.31 | -75.30 to 103.9 | No | ns | >0.9999 |
| -70 mV | 1.042 | -88.56 to 90.65 | No | ns | >0.9999 |
| -60 mV | -18.69 | -108.3 to 70.91 | No | ns | 0.9997 |
| -50 mV | -37.53 | -127.1 to 52.07 | No | ns | 0.931 |
| -40 mV | -61.59 | -151.2 to 28.02 | No | ns | 0.4155 |
| -30 mV | -92.93 | -182.5 to -3.331 | Yes | * | 0.0367 |
|  |  |  |  |  |  |
| Source of Variation | % of total variation | P value | P value summary | Significant? |  |
| Interaction | 3.108 | 0.0068 | ** | Yes |  |
| Row Factor | 81.52 | <0.0001 | **** | Yes |  |
| Column Factor | 0.001047 | 0.9281 | ns | No |  |
|  |  |  |  |  |  |
| ANOVA table | SS | DF | MS | F (DFn, DFd) | P value |
| Interaction | 83827 | 9 | 9314 | F (9, 120) = 2.696 | P=0.0068 |
| Row Factor | 2198498 | 9 | 244278 | F (9, 120) = 70.71 | P<0.0001 |
| Column Factor | 28.25 | 1 | 28.25 | F (1, 120) = 0.008177 | P=0.9281 |
| Residual | 414546 | 120 | 3455 |  |  |
|  |  |  |  |  |  |
| Difference between column means |  |  |  |  |  |
| Mean of ARS | -56.1 |  |  |  |  |
| Mean of ARS+HFTS | -55.2 |  |  |  |  |
| Difference between means | -0.8984 |  |  |  |  |
| SE of difference | 9.935 |  |  |  |  |
| 95% CI of difference | -20.57 to 18.77 |  |  |  |  |
|  |  |  |  |  |  |
| Data summary |  |  |  |  |  |
| Number of columns (Column Factor) | 2 |  |  |  |  |
| Number of rows (Row Factor) | 10 |  |  |  |  |
| Number of values | 140 |  |  |  |  |

| **Table S10.** The K2P current to different voltage in PNs of ARS mice before and after BLS (ARS vs. ARS+BLS mice) | | | | | |
| --- | --- | --- | --- | --- | --- |
|  |  |  |  | Related to Figure7 |  |
| ARS - ARS+BLS | Mean Diff. | 95.00% CI of diff. | Below threshold? | Summary | Adjusted P Value |
| -120 mV | 27.66 | -47.06 to 102.4 | No | ns | 0.9696 |
| -110 mV | 25.26 | -49.46 to 99.97 | No | ns | 0.984 |
| -100 mV | 17.84 | -56.87 to 92.56 | No | ns | 0.999 |
| -90 mV | 10.98 | -63.73 to 85.70 | No | ns | >0.9999 |
| -80 mV | 4.272 | -70.44 to 78.99 | No | ns | >0.9999 |
| -70 mV | 0.3105 | -74.40 to 75.03 | No | ns | >0.9999 |
| -60 mV | -4.102 | -78.82 to 70.61 | No | ns | >0.9999 |
| -50 mV | -7.782 | -82.50 to 66.93 | No | ns | >0.9999 |
| -40 mV | -8.752 | -83.47 to 65.96 | No | ns | >0.9999 |
| -30 mV | -7.242 | -81.96 to 67.47 | No | ns | >0.9999 |
|  |  |  |  |  |  |
| Source of Variation | % of total variation | P value | P value summary | Significant? |  |
| Interaction | 0.294 | 0.9802 | ns | No |  |
| Row Factor | 80.76 | <0.0001 | **** | Yes |  |
| Column Factor | 0.05822 | 0.4836 | ns | No |  |
|  |  |  |  |  |  |
| ANOVA table | SS | DF | MS | F (DFn, DFd) | P value |
| Interaction | 7763 | 9 | 862.5 | F (9, 160) = 0.2767 | P=0.9802 |
| Row Factor | 2132156 | 9 | 236906 | F (9, 160) = 76.00 | P<0.0001 |
| Column Factor | 1537 | 1 | 1537 | F (1, 160) = 0.4931 | P=0.4836 |
| Residual | 498754 | 160 | 3117 |  |  |
|  |  |  |  |  |  |
| Difference between column means |  |  |  |  |  |
| Mean of ARS | -59.01 |  |  |  |  |
| Mean of ARS+BLS | -64.85 |  |  |  |  |
| Difference between means | 5.844 |  |  |  |  |
| SE of difference | 8.323 |  |  |  |  |
| 95% CI of difference | -10.59 to 22.28 |  |  |  |  |
|  |  |  |  |  |  |
| Data summary |  |  |  |  |  |
| Number of columns (Column Factor) | 2 |  |  |  |  |
| Number of rows (Row Factor) | 10 |  |  |  |  |
| Number of values | 180 |  |  |  |  |
